# Analgesic opioids in pregnancy and placental malperfusion-related disorders: a population-based cohort study

**DOI:** 10.1093/ije/dyaf137

**Published:** 2025-08-06

**Authors:** Jonathan Brett, Claudia Bruno, Bianca Varney, Alys Havard, Antonia Shand, Krista F Huybrechts, Helga Zoega

**Affiliations:** Faculty of Medicine and Health, St Vincent’s Clinical School, University of New South Wales, Sydney, Australia; Faculty of Medicine and Health, School of Population Health, University of New South Wales, Sydney, Australia; Faculty of Medicine and Health, School of Population Health, University of New South Wales, Sydney, Australia; Faculty of Medicine and Health, School of Population Health, University of New South Wales, Sydney, Australia; Faculty of Medicine and Health, School of Population Health, University of New South Wales, Sydney, Australia; National Drug and Alcohol Research Centre, University of New South Wales, Sydney, Australia; Maternal and Fetal Medicine, Royal Hospital for Women, Sydney, Australia; Faculty of Medicine and Health, Leeder Centre for Health Policy, Economics, and Data, Child Population and Translational Health Research, The University of Sydney, Sydney, Australia; Division of Pharmacoepidemiology and Pharmacoeconomics, Department of Medicine, Brigham and Women’s Hospital and Harvard Medical School, Boston, MA, United States; Faculty of Medicine, Centre of Public Health Sciences, University of Iceland, Iceland

**Keywords:** opioid, analgesic, pregnancy, ischaemic placental disease, placental malperfusion, small for gestational age, preterm, pre-eclampsia, placental abruption

## Abstract

**Background:**

Analgesic opioid use in pregnancy could increase the risk of disorders related to placental malperfusion, but this relationship is incompletely characterized. We aimed to study the causal association between analgesic opioids in pregnancy and placental abruption, pre-eclampsia, preterm birth, and fetal growth restriction (FGR).

**Methods:**

We conducted a population-based cohort study of pregnancies resulting in birth at ≥20 weeks of gestation between July 2013 and December 2019 in New South Wales, Australia. Linked data on pregnancy, births, medication dispensation, and health services were used. Opioid exposure was defined as at least one opioid dispensation from the last menstrual period to birth. We stratified analyses by exposure in early (≤20 weeks) and/or late (>20 weeks) pregnancy and opioid type by using non-exposed pregnancies as a comparator. We estimated risks by using Cox proportional-hazards models and time-varying exposure, adjusting for demographics, comorbidities, and other medications.

**Results:**

Among 509 971 births, 32 266 (6.3%) had an opioid dispensation. We observed modestly increased risks with any opioid exposure for placental abruption [adjusted hazard ratio (HR) 1.22, 95% confidence interval (CI) 1.06–1.41] and preterm birth (adjusted HR 1.23, 95% CI 1.18–1.28), but not for pre-eclampsia (adjusted HR 1.06, 95% CI 0.99–1.13) or FGR (adjusted HR 0.95, 95% CI 0.88–1.02). Risks of abruption were the most elevated when exposure occurred in both early and late pregnancy (adjusted HR 1.76, 95% CI 1.30–2.40) and for preterm birth when exposure occurred in late-only pregnancy (adjusted HR 1.36, 95% CI 1.27–1.45). Monotherapy with both codeine and oxycodone was associated with elevated risks of abruption and preterm birth.

**Conclusion:**

In this population-based cohort study, we observed modestly increased risks of preterm birth and placental abruption after analgesic opioid use in pregnancy, driven by codeine and oxycodone—the two most frequently used opioids.

Key Messages

- We investigated whether analgesic opioid use during pregnancy is associated with increased risks of placental abruption, pre-eclampsia, preterm birth, and fetal growth restriction (FGR).
- Opioid use in pregnancy was associated with modestly increased risks of placental abruption and preterm birth, particularly with codeine and oxycodone exposure, but not with pre-eclampsia or FGR.
- These findings inform clinicians and patients about specific pregnancy risks related to opioid analgesic use, aiding safer prescribing and risk counselling during pregnancy.

## Introduction

Pain in pregnancy is common, with low back pain and pelvic pain reported in >70% of pregnancies [1]. Opioids, including codeine, tramadol, and oxycodone, are often used to treat pain in pregnancy, with a prevalence ranging from 4 to 191 per 1000 [2]. While the prevalence of analgesic opioid use in pregnancy appears stable or decreasing in most countries, it continues to increase in others such as Iceland, the UK, New Zealand, and Norway [2], raising concerns about potential harms to mother and baby from antenatal exposure.

Adverse perinatal outcomes may share a common aetiology due to placental insufficiency and malperfusion [3, 4], including pre-eclampsia, placental abruption, fetal growth restriction (FGR), and preterm birth. These conditions are leading causes of perinatal morbidity and mortality, and may reoccur in subsequent pregnancies. Pre-eclampsia, typically marked by new-onset hypertension and end-organ involvement after 20 weeks, occurs in 2%–10% of pregnancies, depending on the population and definitions used [6, 7]. Placental abruption, defined as partial or complete separation of the placenta from the uterus, has a prevalence of 0.4%–1% [5]. FGR and preterm birth (including spontaneous and iatrogenic) are estimated to affect 3%–5% [5] and 5%–13% [8] of pregnancies, respectively. Due to the amount of information required for a diagnosis of FGR, low birthweight is often used as a proxy, as described below.

While the causes of poor placentation are incompletely understood, poor trophoblast invasion and incomplete remodelling of spiral arteries during placentation are considered important mechanistic factors leading to uteroplacental under-perfusion, chronic hypoxia, and placental ischaemia [9]. Opioids are known to cross the placenta and have the potential to cause fetal and placental harm. Animal studies support the hypothetical association between *in utero* opioid exposure and placental malperfusion [3, 5]. Kappa opioid receptor agonism has been found to interfere with acetylcholine-facilitated amino acid transport, which is essential for spiral artery remodelling [10]. Several observational studies in humans have investigated associations with analgesic opioid exposure in pregnancy and disorders related to placental malperfusion, but demonstrate inconsistent results [16]. Contributing to this inconsistency are small study sample sizes, uncertainties around the period of opioid exposure that is critical to affecting placentation, and whether risks vary by specific opioid owing to variations in clinical pharmacology.

With this study, we aimed to investigate whether *in utero* exposure to prescription opioid analgesics increases the risk of adverse pregnancy outcomes related to placental malperfusion. Specifically, we aimed to identify whether *in utero* exposure to any prescription opioid analgesic or specific opioids codeine and oxycodone in early- and/or late-pregnancy results in an increased risk of pre-eclampsia, placental abruption, FGR, and preterm birth.

## Methods

### Setting

Australia has a publicly funded universal healthcare system in which hospitalizations, antenatal care, outpatient family medicine, and specialist care and prescription medications are government-subsidized. This study leveraged the Early Life Course data platform [17], which comprises multiple routine data collections on health, education, and social services for all children born in New South Wales (NSW) since 2001, their mothers, and second parents. NSW is Australia’s most populous state, with almost 100 000 births annually, accounting for a third of all births in Australia each year [17]. For this study, information on pregnancies resulting in birth was ascertained from the NSW Perinatal Data Collection, which is mandatory data collection containing information on all births, including stillbirths, of ≥20 weeks of gestation or birthweight of 400 g in NSW public and private hospitals and home births. Dispensation claim records included all medications dispensed in the community or private hospitals in NSW.

### Study population

We included all births to women residing in NSW, Australia, whose date of last menstrual period (LMP) occurred between 1 July 2013 and 27 March 2019. To limit the population to women who were using opioids for analgesic purposes, we excluded pregnancies amongst women with a history of opioid-use disorder 12 months before the start of pregnancy through to birth (Supplementary Figure S1 and Supplementary Table S1).

### Exposure

We considered all analgesic opioids available through public subsidy in Australia during the period of observation (Supplementary Table S2). We assessed analgesic opioid exposure as at least one dispensation during the following periods: ‘anytime in pregnancy’, defined as LMP (calculated as date of birth minus best clinical estimate of gestational age) to birth minus 1 day; ‘early pregnancy’, defined as LMP to the 20th week of gestation regardless of later exposure; ‘late pregnancy’ but not early pregnancy (‘late-only pregnancy’), defined as after the 20th week of gestation to birth minus 1 day; and both early and late pregnancy.

We assessed monotherapy exposure to codeine-only and oxycodone-containing products (including combination products) separately (Supplementary Table S2), as these are the two most prevalent opioids dispensed during pregnancy in NSW [2].

We compared pregnancies among women exposed to opioids at any point in pregnancy, late-only pregnancy, and both early and late pregnancy to pregnancies among women with no opioid exposure from 90 days before pregnancy through to birth. For early-pregnancy exposure, we compared with pregnancies among women with no opioid exposure from 90 days before pregnancy to the end of early pregnancy (20th week of gestation) to ensure that the exposure ascertainment windows were aligned between the exposed and the referent group.

### Outcomes

The outcomes of interest were recorded in the Perinatal Data Collection (via check boxes) at birth or identified by using the International Classification of Diseases 10th Revision (ICD-10-AM) [18] diagnosis codes in the Admitted Patient Data Collection relating to admissions at any time during pregnancy. We included pre-eclampsia (ICD-10-AM: O11, O14), placental abruption (ICD-10-AM: O45), preterm birth defined as a gestational age of <37 weeks at birth (including spontaneous and iatrogenic), and FGR, defined here as a birthweight less than the third centile for gestational age and sex according to Australian birthweight centiles [19]. The date of onset of placental abruption and pre-eclampsia was defined as the admission date for the first relevant hospitalization when recorded in the Admitted Patient Data Collection. An additional 10% of cases of pre-eclampsia were detected only in Perinatal Data Collection and, in this instance, the date of birth was used as the date of onset. If a pregnancy was found to have more than one type of disorder related to placental malperfusion, then each of these was considered to have occurred and we analysed the risks of each as non-mutually exclusive outcomes.

### Covariates

As covariates, we included maternal characteristics, namely age, maternal birth country [20], marital status, health insurance, socioeconomic status of residential area based on the Index of Relative Socio-economic Disadvantage grouped into quintiles [21], remoteness based on the Accessibility/Remoteness Index of Australia [22], smoking status throughout pregnancy, and maternal healthcare utilization (Supplementary Table S3). In addition, we identified maternal health conditions in the 12 months before pregnancy throughout pregnancy except for conditions that could have been caused by or arisen during pregnancy, for which we used the 12 months prior to pregnancy and not pregnancy to identify these conditions (Supplementary Table S3). We also included the calendar year of LMP, parity, whether the pregnancy was multifetal and prior diagnoses of placental abruption, pre-eclampsia, preterm birth, or FGR. For covariates with <5% missing, we imputed the median value of that covariate. Body mass index (BMI) was the only covariate with >5% missing and is discussed below.

### Data analysis

We summarized maternal and pregnancy characteristics according to the opioid-exposure status during different pregnancy periods. We estimated the crude cumulative incidence of outcomes by opioid-exposure status using Kaplan–Meier survival curves. To determine the association between opioid exposure in each pregnancy period and each outcome of interest, we calculated hazard ratios (HRs) by using Cox proportional hazard models with time-varying exposure [23]. We used gestational age as the underlying timescale for the analyses, opioid exposure was updated on a weekly basis from the start of pregnancy, and follow-up was censored at the occurrence of the outcome or birth. For pregnancies with both early and late opioid exposure, we considered pregnancies exposed at the time of the first dispensation in the late-pregnancy period. For preterm birth, we censored follow-up at 37 weeks, as pregnancies were no longer at risk of the outcome. For all analyses, the unit of analysis was the pregnancy, except for FGR, for which it was the infant. We calculated HRs, unadjusted and adjusted for all covariates described above, and estimated robust 95% confidence intervals (CIs), accounting for correlation relating to individual women with more than one observed pregnancy. Schoenfeld residual plots were visually examined to assess the proportional-hazards assumption. All analyses were conducted by using RStudio (Version 2024.04.2, R v4.4.1).

### Sensitivity analyses

First, to assess potential exposure misclassification, we defined opioid exposure as requiring two opioid dispensations, with exposure starting at the date of the second dispensation. Second, to evaluate the role of multifetal pregnancy and parity on the observed associations, we restricted the cohort to the following subpopulations: singleton pregnancies, nulliparous women (first birth only), and parous (any births after first births). Third, to evaluate the impact of opioid exposure from dispensations occurring before pregnancy start, we extended the exposure window to include opioid exposures 90 days before the LMP. Fourth, to evaluate the impact of unmeasured confounders, we altered the comparison group to pregnancies among women with an opioid exposure prior to pregnancy defined as one or more opioid dispensations in the 12 months leading to 90 days before the LMP but none thereafter (opioid discontinuers). Fifth, we only considered maternal conditions recorded before pregnancy (and not during) as covariates, except for teratogen use. BMI was not routinely collected prior to 2016 and so, sixth, we performed a sensitivity analysis including BMI as a covariate by using data collected from 2016 onwards. Lastly, as low birthweight was a proxy for FGR and was only recorded at birth, we repeated the primary analysis for FGR by using multiple variable Poisson regressions rather than time-to-event analyses.

To determine the robustness of the identified associations to potential unmeasured confounding, we calculated the E-value [24] to quantify the amount of unmeasured confounding that would be required to account for the observed associations.

## Results

Overall, we identified 509 971 eligible pregnancies, of which 32 266 (6.3%) had an opioid dispensation: 17 551 (3.4%) in early pregnancy, 14 715 (2.8%) in late-only, and 3680 (0.7%) in both early and late pregnancy (Table 1). Women who were exposed to opioids during pregnancy were more likely to be younger, born in a Western country, parous, and live in an area of higher social disadvantage. They were also more likely to live outside of major cities, smoke during pregnancy, and have a recorded health condition (Table 1). Of pregnancies exposed to opioids, 25 925 (80.3%) and 3234 (10.6%) were exposed to codeine and oxycodone monotherapy, respectively.

**Table 1.** Characteristics of pregnancies by timing of exposure to opioids.

	Any time during pregnancy		Early pregnancy		Late pregnancy only	Both early and late pregnancy
	Unexposed *N* = 477 705 [*n* (%)]	Exposed *N* = 32 266 [*n* (%)]	Unexposed *N* = 492 420 [*n* (%)]	Exposed *N* = 17 551 [*n* (%)]	Exposed *N* = 14 715 [*n* (%)]	Exposed *N* = 3680 [*n* (%)]
Year of birth^a^						
2013–14	64 672 (14)	4309 (13)	66 768 (14)	2213 (13)	2096 (14)	475 (13)
2015–16	168 697 (35)	11 662 (36)	174 124 (35)	6235 (36)	5427 (37)	1205 (33)
2017–19	244 336 (51)	16 295 (51)	251 528 (51)	9103 (52)	7192 (49)	2000 (54)
Maternal age at conception (years)						
≤19	14 630 (3.1)	1288 (4.0)	15 278 (3.1)	640 (3.6)	648 (4.4)	118 (3.2)
20–24	59 822 (13)	5739 (18)	62 302 (13)	3259 (19)	2480 (17)	696 (19)
25–29	137 387 (29)	9383 (29)	141 703 (29)	5067 (29)	4316 (29)	1070 (29)
30–34	168 509 (35)	9878 (31)	173 165 (35)	5222 (30)	4656 (32)	1032 (28)
35–39	81 245 (17)	4917 (15)	83 387 (17)	2775 (16)	2142 (15)	633 (17)
≥40	16 109 (3.4)	1061 (3.3)	16 582 (3.4)	588 (3.4)	473 (3.2)	131 (3.6)
Missing	<6 (<0.1)	0 (0)	<6 (<0.1)	0 (0)	0 (0)	0 (0)
Parity, number of previous births						
Zero	208 893 (44)	11 681 (36)	214 423 (44)	6151 (35)	5530 (38)	943 (26)
1	166 986 (35)	10 605 (33)	172 003 (35)	5588 (32)	5017 (34)	1112 (30)
2	66 002 (14)	5602 (17)	68 453 (14)	3151 (18)	2451 (17)	827 (22)
≥3	35 715 (7.5)	4369 (14)	37 429 (7.6)	2655 (15)	1714 (12)	797 (22)
Missing	109 (<0.1)	9 (<0.1)	112 (<0.1)	6 (<0.1)	<6 (<0.1)	<6 (<0.1)
Gestational age, weeks						
≤32	7168 (1.5)	528 (1.6)	7283 (1.5)	413 (2.4)	115 (0.8)	66 (1.8)
33–36	24 170 (5.1)	2205 (6.8)	25 045 (5.1)	1330 (7.6)	875 (5.9)	351 (9.5)
≥37	446 367 (93)	29 533 (92)	460 092 (93)	15 808 (90)	13 725 (93)	3263 (89)
Multifetal	6729 (1.4)	572 (1.8)	6992 (1.4)	309 (1.8)	263 (1.8)	64 (1.7)
Missing	20 (<0.1)	<6 (<0.1)	21 (<0.1)	<6 (<0.1)	<6 (<0.1)	0 (0)
Maternal birth country (Western)	318 077 (67)	26 460 (82)	330 023 (67)	14 514 (83)	11 946 (81)	3280 (89)
Marital status (married)	411 711 (86)	24 608 (76)	423 292 (86)	13 027 (74)	11 581 (79)	2535 (69)
Missing	3316 (0.7)	215 (0.7)	3401 (0.7)	130 (0.7)	85 (0.6)	21 (0.6)
Private health insurance	164 139 (34)	8090 (25)	168 040 (34)	4189 (24)	3901 (27)	701 (19)
Missing	2384 (0.5)	192 (0.6)	2461 (0.5)	115 (0.7)	77 (0.5)	21 (0.6)
Quintiles of socioeconomic disadvantage						
1 (high disadvantage)	101 259 (21)	8807 (27)	105 004 (21)	5062 (29)	3745 (25)	1156 (31)
2	75 677 (16)	6631 (21)	78 610 (16)	3698 (21)	2933 (20)	876 (24)
3	95 652 (20)	6259 (19)	98 569 (20)	3342 (19)	2917 (20)	715 (19)
4	93 502 (20)	5470 (17)	96 075 (20)	2897 (17)	2573 (17)	529 (14)
5 (lowest disadvantage)	106 556 (22)	4838 (15)	108 988 (22)	2406 (14)	2432 (17)	375 (10)
Missing	5059 (1.1)	261 (0.8)	5174 (1.1)	146 (0.8)	115 (0.8)	29 (0.8)
Resides in major city	366 384 (77)	22 083 (68)	376 515 (76)	11 952 (68)	10 131 (69)	2361 (64)
Missing	5059 (1.1)	261 (0.8)	5174 (1.1)	146 (0.8)	115 (0.8)	29 (0.8)
BMI						
<18.5	14 880 (3.1)	780 (2.4)	15 231 (3.1)	429 (2.4)	351 (2.4)	89 (2.4)
18.5–24.9	173 229 (36)	8934 (28)	177 358 (36)	4805 (27)	4129 (28)	872 (24)
25–29.9	75 807 (16)	5637 (17)	78 404 (16)	3040 (17)	2597 (18)	669 (18)
≥30	54 208 (11)	6037 (19)	56 651 (12)	3594 (20)	2443 (17)	888 (24)
Missing	159 581 (33)	10 878 (34)	164 776 (33)	5683 (32)	5195 (35)	1162 (32)
Smoking in pregnancy	40 155 (8.4)	6586 (20)	42 650 (8.7)	4091 (23)	2495 (17)	1221 (33)
Missing	233 (<0.1)	24 (<0.1)	244 (<0.1)	13 (<0.1)	11 (<0.1)	<6 (<0.1)
Number of admissions in 12 months before pregnancy						
Zero	447 599 (94)	29 097 (90)	461 210 (94)	15 486 (88)	13 611 (92)	3131 (85)
1	16 245 (3.4)	1552 (4.8)	16 833 (3.4)	964 (5.5)	588 (4.0)	221 (6.0)
2	7117 (1.5)	779 (2.4)	7391 (1.5)	505 (2.9)	274 (1.9)	140 (3.8)
≥3	6744 (1.4)	838 (2.6)	6986 (1.4)	596 (3.4)	242 (1.6)	188 (5.1)
Prior birth outcomes						
Preterm birth	8072 (1.7)	757 (2.3)	8420 (1.7)	409 (2.3)	348 (2.4)	113 (3.1)
Pre-eclampsia	3471 (0.7)	295 (0.9)	3609 (0.7)	157 (0.9)	138 (0.9)	46 (1.3)
Placental abruption	677 (0.1)	61 (0.2)	703 (0.1)	35 (0.2)	26 (0.2)	12 (0.3)
Fetal growth restriction	3177 (0.7)	229 (0.7)	3267 (0.7)	139 (0.8)	90 (0.6)	23 (0.6)
Caesarean birth	76 860 (16)	6179 (19)	79 653 (16)	3386 (19)	2793 (19)	913 (25)
Missing	589 (0.1)	39 (0.1)	605 (0.1)	23 (0.1)	16 (0.1)	<6 (<0.1)
Maternal comorbidities^b^						
Hypertension	7319 (1.5)	919 (2.8)	7673 (1.6)	565 (3.2)	354 (2.4)	164 (4.5)
Diabetes	18 485 (3.9)	1994 (6.2)	19 262 (3.9)	1217 (6.9)	777 (5.3)	314 (8.5)
Cardiovascular disease	24 396 (5.1)	2924 (9.1)	25 588 (5.2)	1732 (9.9)	1192 (8.1)	529 (14)
Chronic renal disease	2707 (0.6)	572 (1.8)	2969 (0.6)	310 (1.8)	262 (1.8)	110 (3.0)
Haematological disease	30 722 (6.4)	3388 (11)	32 178 (6.5)	1932 (11)	1456 (9.9)	521 (14)
Inflammatory/painful conditions	48 217 (10)	8144 (25)	51 129 (10)	5232 (30)	2912 (20)	1563 (42)
Drug and alcohol conditions	3237 (0.7)	578 (1.8)	3430 (0.7)	385 (2.2)	193 (1.3)	128 (3.5)
Mental health conditions	89 986 (19)	12 622 (39)	95 260 (19)	7348 (42)	5274 (36)	2204 (60)
Thyroid conditions	19 162 (4.0)	1340 (4.2)	19 750 (4.0)	752 (4.3)	588 (4.0)	147 (4.0)
Epilepsy	3456 (0.7)	726 (2.3)	3720 (0.8)	462 (2.6)	264 (1.8)	196 (5.3)
Infertility	15 796 (3.3)	1328 (4.1)	16 246 (3.3)	878 (5.0)	450 (3.1)	140 (3.8)
Obesity	13 708 (2.9)	1961 (6.1)	14 448 (2.9)	1221 (7.0)	740 (5.0)	340 (9.2)
Teratogen use in early pregnancy	34 164 (7.2)	4759 (15)	35 840 (7.3)	3083 (18)	1676 (11)	914 (25)

a Note that the study period is based on pregnancies with a LMP between 1 July 2013 and 27 March 2019.

b See Supplementary Table S3 for more information.

**Figure 1.**
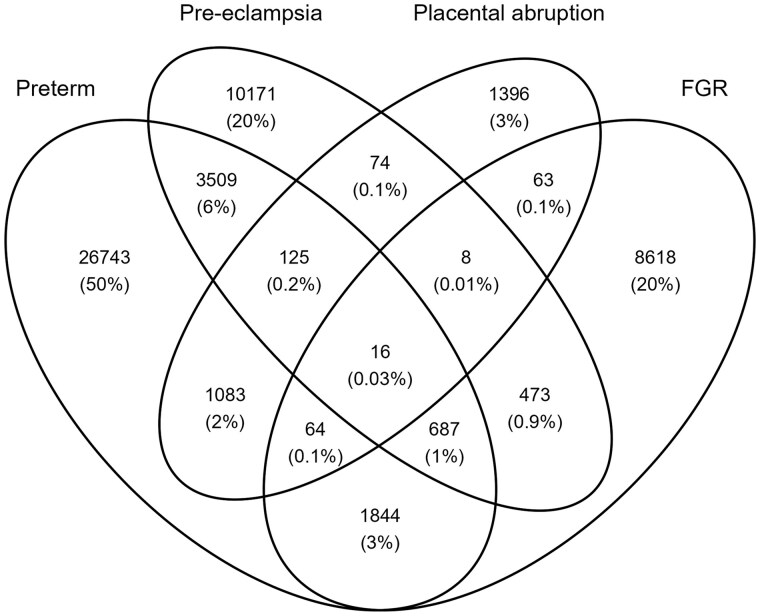
Co-occurrence of pre-eclampsia, placental abruption, preterm birth, and FGR. Number of pregnancies with these outcomes as a percentage of pregnancies with at least one of these outcomes.

Placental abruption occurred in 2829 (0.6%) of pregnancies, pre-eclampsia in 15 063 (3.0%), FGR in 11 773 (2.3%) (11 919 babies), and preterm birth in 34 071 (6.7%). The co-occurrence of these outcomes is shown in Figure 1 and cumulative incidence curves for these outcomes are shown in Supplementary Figure S2.

**Figure 2.**
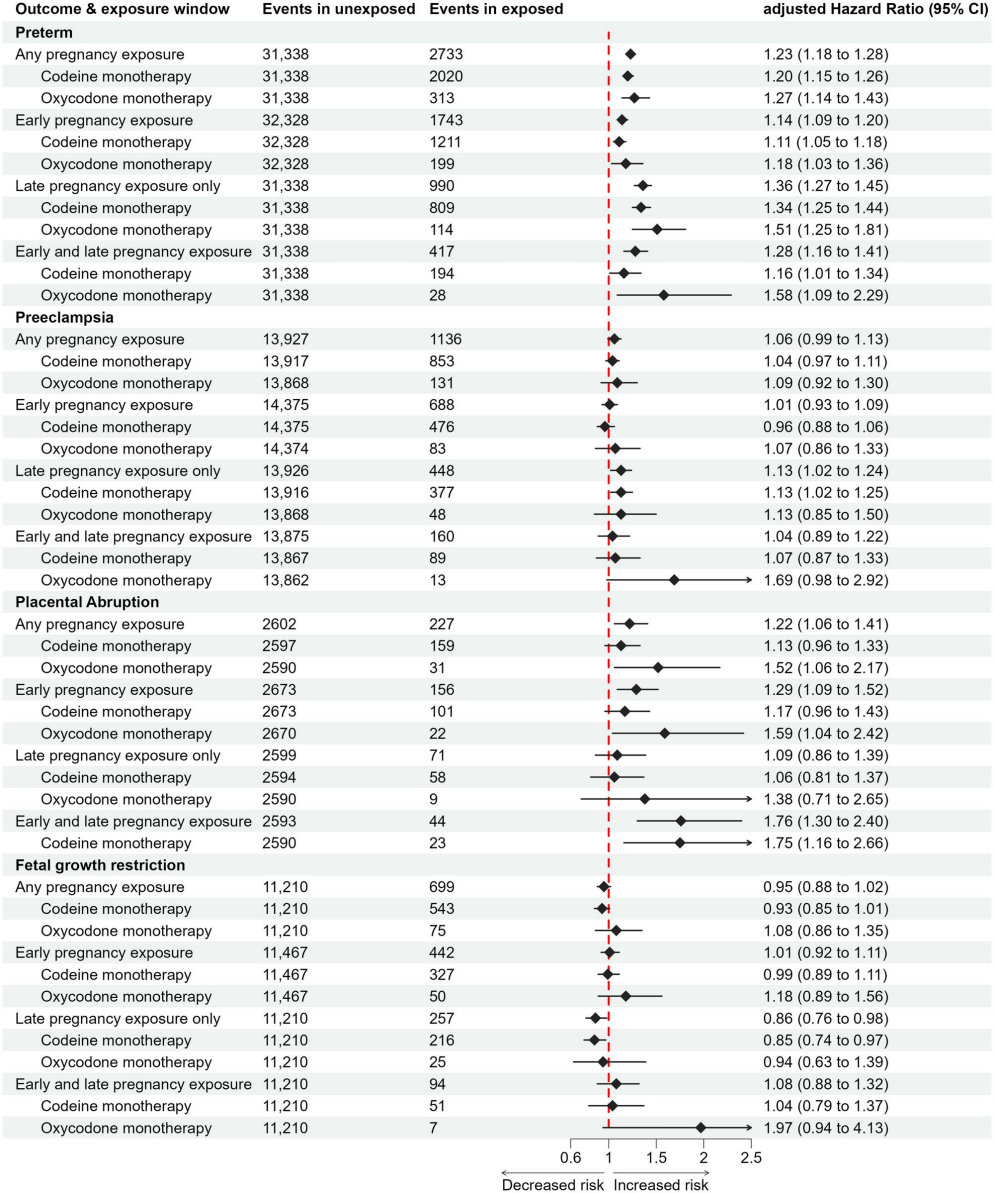
Risk of pre-eclampsia, placental abruption, preterm birth, and FGR by opioid type and timing of exposure. *Oxycodone censored from placental abruption early- and late-pregnancy exposure, as fewer than five events occurred.

HRs for the association between opioid exposure and each outcome of interest attenuated in all analyses after adjustment for available covariates (Figure 2 and Supplementary Table S4). We observed modestly increased risks for placental abruption (adjusted HR 1.22, 95% CI 1.06–1.41) with opioid exposure anytime in pregnancy and this association was strongest for early-only pregnancy exposure and exposure in both early and late pregnancy (Figure 2 and Supplementary Table S4). Risk of preterm birth with exposure anytime in pregnancy exposure was also modestly increased (Adjusted Hazard Ratio (aHR) 1.23, 95% CI 1.18–1.28) and this association was strongest for late-only pregnancy exposure and both early and late exposure (Figure 2 and Supplementary Table S4). There were no associations with opioid exposure and pre-eclampsia or FGR following opioid exposure anytime in pregnancy or by specific pregnancy periods (Figure 2 and Supplementary Table S4). With opioid monotherapy, the risk of placental abruption was increased the most with oxycodone exposure in early pregnancy and codeine exposure in both early and late pregnancy. Risk of preterm birth was increased the most with both late-only and early- and late-pregnancy exposures to codeine and oxycodone (Figure 2 and Supplementary Table S4).

### Sensitivity analyses

Results were generally robust to sensitivity analyses, with some exceptions (Supplementary Tables S5–S14). Effect sizes were generally greater when the exposure required two dispensations and the risk of pre-eclampsia increased when restricting to non-first pregnancies, when restricting to covariates measured only before pregnancy and when using opioid discontinuers as comparators. The calculated E-score suggested that the observed risk for placental abruption associated with opioid exposure in both early and late pregnancy would require an unmeasured confounding effect size of 2.9 to explain the observed association. Similarly, an unmeasured confounding effect size of 2.0 would explain the observed association of preterm birth with late-only opioid pregnancy exposure, indicating that a strong and greater-than-expected effect of unmeasured confounding would be required to account for these observed associations.

## Discussion

We present a population-based analysis of analgesic opioids and adverse pregnancy outcomes in over half a million pregnancies in NSW, Australia. While opioid use was not associated with FGR or pre-eclampsia, we observed a 22% (6%–41%) increased risk for placental abruption, mainly with opioid use in early and both early and late pregnancy, and a 23% (18%–28%) increased risk for preterm birth, mainly with opioid use in late-only and both early and late pregnancy. These increased risks were likely driven by codeine and oxycodone—the two most-used opioids in our cohort.

Our findings were generally consistent with results from a US cohort study by Esposito *et al.*, except that we did not find an increased risk of FGR with opioid exposure [12]. Differences in exposure and outcome definitions may explain this, though other studies have also not found associations between opioid exposure and FGR [13, 14, 16]. Consistently with existing knowledge [5], we identified a substantial co-occurrence of outcomes related to placental malperfusion, more commonly in preterm than term gestations [5].

Prior studies have also demonstrated associations between opioid exposure and placental abruption, with effect sizes ranging from 30% to 40% increased risk [12, 13]. This risk was associated with early-pregnancy use [12] and may be related to the disruption of a critical period of placentation in early pregnancy. Although placental abruption is painful, raising the possibility of protopathic bias, the higher risk with early-pregnancy use and when two dispensations were required to define exposure makes this less likely.

Similarly, previous studies have consistently found an increased risk of preterm birth with opioid exposure, with effect sizes ranging from 12% to 30% [13, 16, 25]. While pre-eclampsia and FGR are common reasons for planned preterm birth, spontaneous preterm birth may also be associated with unmeasured potential confounders such as infection or inflammation associated with preterm premature rupture of the membranes and uterine overdistension due to polyhydramnios [8].

Neither we nor Esposito *et al.* [12] observed an increased risk of pre-eclampsia, though several other studies have. A Swedish population-based study combining self-reported early-pregnancy opioid use with prescription records in late pregnancy reported an increased risk for pre-eclampsia [1.34 odds ratio (OR), 95%CI 1.24–1.44] [13]. Similarly, a Finnish study using self-reported opioid exposure found an increased risk for mild (OR 2.94, 95% CI 1.66–2.98) but not severe pre-eclampsia [15]. However, these two studies included women receiving opioids as part of opioid-agonist therapy for opioid-use disorder. Methodological differences and residual confounding may explain these inconsistencies.

Our findings that both codeine monotherapy and oxycodone monotherapy were associated with increased risks of placental abruption and preterm birth, albeit with wide CIs, are novel and salient given that codeine and oxycodone are the most prevalent opioids used in pregnancy in Australia and many other countries [2].

The modest increases in risks for placental abruption and preterm birth observed in this study should be interpreted in the context of the clinical need to treat uncontrolled pain in this population with relatively few treatment options. Due to resource and ethical considerations, there is little prospect of confirming these findings with randomized–controlled trials. Hence, consistent findings from robust observational studies should be used to initiate conversations with pregnant women about the relative benefits and risks of opioid use in pregnancy.

While we were able to reliably determine opioid dispensations and specific perinatal outcomes and conduct time-varying analyses to reduce the risk of time-related bias, our study was subject to several limitations. First, dispensation records do not reflect actual consumption. However, the directions of association of our findings were robust in sensitivity analyses requiring two opioid dispensations to define exposure—a context in which opioid consumption was more likely to have occurred. Second, FGR was based on a proxy of small for gestational age captured by weight at birth; hence, it is possible that problems with fetal growth might have started prior to opioid use. Third, restriction of the cohort to pregnancies resulting in live births implies that we are estimating the direct effect of opioids on the outcome of interest based on the assumption that opioids do not affect the risk of early-pregnancy loss or maternal death, and that there are no uncontrolled common causes of these events and the outcomes of interest. Finally, there may be residual confounding, particularly relating to tobacco smoking and alcohol exposure, the family home environment, and chronic painful conditions. However, the relatively large E-value and the robustness of the findings in our sensitivity analyses strengthen our case that we have adjusted for the most important confounders.

## Conclusion

Here, we find a modest increased risk of placental abruption and preterm birth with opioid exposure in pregnancy and specifically with codeine monotherapy and oxycodone monotherapy. These findings are consistent with those of prior observational studies and should be used to inform the risk–benefit assessment of analgesic opioid use in pregnancy.

## Ethics approval

Ethics approval for the Early Life Course data platform was granted by the NSW Population and Health Services Research Ethics Committee (PHSREC) (2019/ETH11830), the Australian Institute of Health and Welfare Ethics Committee (EC 2020/2/1130), and the Aboriginal Health and Medical Research Council of NSW Ethics Committee (1689/20).

## Supplementary Material

dyaf137_Supplementary_Data

## Data Availability

Data cannot be shared, for ethical/privacy reasons.
