# Supplementary material for: Analgesic opioids in pregnancy and placental malperfusion-related disorders: a population-based cohort study
Source: Int J Epidemiol. 2025 Aug 6;54(4):dyaf137. doi: 10.1093/ije/dyaf137 (PMC12342150; doi:10.1093/ije/dyaf137)
Supplement: dyaf137_Supplementary_Data [file dyaf137_supplementary_data.docx]

**Supplementary material**

[Figure S1: Study population and exclusion criteria 2](#_Toc187420142)

[Table S1: Study population exclusions relating to maternal opioid use disorder 3](#_Toc187420143)

[Table S2: Analgesic opioids included in study 4](#_Toc187420144)

[Table S3: Covariates 5](#_Toc187420145)

[Figure S2: Crude cumulative incidence curves for each outcome by exposure to analgesic opioids 8](#_Toc187420146)

[Table S4: Risk of preeclampsia, placental abruption, preterm birth and fetal growth restriction by opioid type of opioid and timing of exposure 9](#_Toc187420147)

[Table S5: Risk of preeclampsia, placental abruption, preterm birth and fetal growth restriction by opioid type of opioid and timing of exposure. Requiring two prescription fills in each pregnancy period. 11](#_Toc187420148)

[Table S6: Risk of preeclampsia, placental abruption, preterm birth and fetal growth restriction by opioid type of opioid and timing of exposure: Restricting study population to singleton births 13](#_Toc187420149)

[Table S7: Risk of preeclampsia, placental abruption, preterm birth and fetal growth restriction by opioid type of opioid and timing of exposure. Restricting to only first pregnancies 15](#_Toc187420150)

[Table S8: Risk of preeclampsia, placental abruption, preterm birth and fetal growth restriction by opioid type of opioid and timing of exposure. Restricting to non-first pregnancies 17](#_Toc187420151)

[Table S9: Risk of preeclampsia, placental abruption, preterm birth and fetal growth restriction by opioid type of opioid and timing of exposure. Restricting covariates included in the regression model to those only measured before pregnancy 19](#_Toc187420152)

[Table S10: Risk of preeclampsia, placental abruption, preterm birth and fetal growth restriction by opioid type of opioid and timing of exposure. Including Body Mass Index (BMI) by restricting analyses to births from 2016 onwards. 21](#_Toc187420153)

[Table S11: Risk of preeclampsia, placental abruption, preterm birth and fetal growth restriction by opioid type of opioid and timing of exposure. Using women who had opioid exposure prior to but not during pregnancy (opioid discontinuers) as comparator. 23](#_Toc187420154)

[Table S12: Risk of preeclampsia, placental abruption, preterm birth and fetal growth restriction by opioid type of opioid and timing of exposure. Including pregnant women with opioid exposure in the 90 days prior to pregnancy but not the first 20 weeks of pregnancy in the exposed group 25](#_Toc187420155)

[Table S13: Poisson regression analysis for risk of fetal growth restriction 26](#_Toc187420156)

Figure S1: Study population and exclusion criteria

| Exclusion reasons | Number of babies | Number of pregnancies |
| --- | --- | --- |
| **All births to NSW residents in NSW^a^** | 540,966 | 533,182 |
| **Overall excluded** | 23,584 | 23,211 |
| **Reason for exclusion:** |  |  |
| *History of Opioid Use Disorder^b^* | 1,535 | 1,515 |
| Opioid Agonist Therapy | 772 | 763 |
| Diagnosis of opioid use disorder or opioid overdose | 1,171 | 1,156 |
| Treatment with naloxone | 10 | 9 |
| *Missing linkage with delivery* | 5,728 | 5,665 |
| One hospital not included in linkage | 1,712 | 1,689 |
| No linkage | 5,281 | 5,223 |
| *Recieved opioids 90days prior & not first 20 weeks* | 16,539 | 16,247 |
| **Remaining study population** | 517,382 | 509,971 |
| ^a^Study period is based on pregnancies with a last menstrual period between 1 July 2013 and 27 March 2019 | | |
| ^b^Recorded in the 12 months prior to start of pregnancy through to birth to ensure more complete capture due to increased health system contacts during pregnancy  NSW, New South Wales | | |

Table S1: Study population exclusions relating to maternal opioid use disorder

| **Exclusion** | **Dataset** |
| --- | --- |
| Pregnancies among women who had one or more dispensings of naloxone (Pharmaceutical Benefits Scheme ATC code: V03AB15) 12 months before last menstrual period (LMP) through to birth.  *Note: this may include some women without opioid use disorder accessing naloxone to treat opioid overdose in others.* | Australian Pharmaceutical Benefits Scheme (dispensing claims) |
| Pregnancies among women with any record of a authority for a opioid (required for drug dependent individuals and certain opioids prescribed for longer than three months)[1] or authority for opioid agonist therapy to treat opioid use disorder 12 months before LMP through to birth. | New South Wales Controlled Drugs Data Collection |
| Pregnancies among women with any diagnosis of opioid use disorder (ICD-10-AM codes: F11.1, F11.2, or opioid overdose (T40.0-T40.2) 12 months before LMP through to birth. | New South Wales Admitted Patient Data Collection |

ATC, Anatomical Therapeutic Chemical Classification; ICD-10-AM, International Classification of Diseases-10-Australian Modification.

Table S2: Analgesic opioids included in study

| **Prescription Opioid** | **ATC code** |
| --- | --- |
| Buprenorphine^c^ | N02AE01 |
| Codeine ^a^ | R05DA04 |
| Codeine and acetylsalicylic acid ^a^ | N02AJ07 |
| Codeine and paracetamol ^a^ | N02AJ06 |
| Fentanyl | N02AB03 |
| Fentanyl |  |
| Hydromorphone | N02AA03 |
|  |  |
| Meperidine/pethidine | N02AB02 |
| Methadone | N02AC52 |
|  |  |
| Morphine | N02AA01 |
|  |  |
| Oxycodone ^b^ | N02AA05 |
| Oxycodone and naloxone ^b^ | N02AA55 |
| Tapentadol | N02AX06 |
| Tramadol | N02AX02 |

^a^ Included in codeine monotherapy analysis,^b^ Included in oxycodone monotherapy

^c^Formulations of buprenorphine and methadone for the treatment of opioid use disorder are not captured in this dataset, hence the formulations included here are for analgesic use only.

ATC, Anatomical Therapeutic Chemical Classification.

Table S3: Covariates

| **Covariate** | **Data collections** | **Time of capture, ICD-10-AM and ATC codes used and notes:** |
| --- | --- | --- |
| **Calendar year of birth**  (Categorical: 2012-2019) | Perinatal registry | **Captured at time of** **birth:**  LMP = birth date – gestational age*7 |
| **Maternal age**  (Categorical: ≤19, 20-24, 25-29, 30-34, 35-39, ≥40) | Perinatal registry | **Captured at time of** **birth:**  Missing values were imputed with median value. |
| **Maternal country of birth**  (Dichotomous: 1= yes, 0 = no) | Perinatal registry | **Captured at birth:**  Coded according to the Australian Bureau of Statistics Standard Australian Classification of Countries (SACC), 2016 [2].  Missing values were imputed with median value. |
| **Marital status**  (Dichotomous: 1= yes, 0 = no) | Hospital records | **Captured at birth:**  Partner (Married including de facto) vs no partner. Missing values were imputed with median value. |
| **Private health insurance**  (Dichotomous: 1= yes, 0 = no) | Hospital records | **Captured at birth:**  Missing values were imputed with median value. |
| **Socio-economic disadvantage**  (Categorical: Q1 to Q5.) | Perinatal registry | **Captured at birth:**  Statistical Local Area (SLA) of residence mapped to 2016 Index of Relative Socio-economic Disadvantage.  Q1 corresponds to highest level of disadvantage, and Q5 the least. Missing values were imputed with median value. |
| **Remoteness of residence** (Categorical: Major city vs other) | Perinatal registry | **Captured at birth:**  Statistical Local Area (SLA) of residence mapped to 2016 Accessibility and Remoteness Index of Australia.  Missing values were imputed with median value. |
| **Body Mass Index (BMI):** (Categorical: <18.5, 18.5-24.9, 25-29.9, ≥30) | Perinatal registry | **Captured at time of birth:**  Based on maternal weight and height as recorded in first trimester of pregnancy. Only used for analysis after 2016. |
| **Obstetric** |  |  |
| **Multiple birth**  (Dichotomous: 1= yes, 0 = no) | Perinatal registry | **Captured at birth** |
| **Parity**  (Categorical: 0, 1, 2, or ≥3 previous births) | Perinatal registry | **Captured at birth:**  Missing values were imputed with median value. |
| **Previous caesarean**  (Dichotomous: 1= yes, 0 = no) | Perinatal registry | **Captured at birth:**  If information on previous caesareans were missing and it was the first birth, then recorded as no. Otherwise missing values were imputed with median value. |
| **Outcome occurred in previous pregnancies** | Perinatal registry | **Any previous pregnancy:**  See outcome definitions for more information. |
| (Dichotomous: 1= yes, 0 = no) | Hospital records | **Any previous pregnancy:**  See outcome definitions for more information. |
| **Maternal comorbidities** |  |  |
| **Number of hospital admissions**  (Categorical: 0, 1, 2, ≥3 admissions) | Hospital records | **Year prior to last menstrual period (LMP):**  Totals were grouped into the following mutually exclusive categories: no previous admissions, 1 prior admission, 2 prior admissions, or 3 or more prior admissions. |
| **Smoking during pregnancy** | Perinatal registry | **Captured at birth:**  Based on registry questionnaire regarding any smoking throughout the pregnancy |
| (Dichotomous: 1= yes, 0 = no) | Hospital records | **Records from LMP to birth:**  Z72.0 |
|  | Pharmaceutical  dispensing data | **Records from LMP to birth:**  N07BA02, N07BA03, N07BA01 |
| **Obesity**  (Dichotomous: 1= yes, 0 = no) | Hospital records | **Records from year prior to LMP to birth:**  E66.0, E66.01, E66.09, E66.1, E66.2, E66.3, E66.8, E66.9 Supplementary: U78.1 |
| **Drug and alcohol disorder**  (Dichotomous: 1= yes, 0 = no) | Hospital records | **Records from year prior to LMP to birth:**  F10, F11, F12, F13, F14, F15, F16, F17 F18, F19, Z50.2, Z50.3, Z72.1, Z72.2 |
|  | Mental Health Ambulatory data | **Records from year prior to LMP to birth:**  F17 |
|  | Pharmaceutical  dispensing data | **Records from year prior to LMP to birth:**  N07BB01 - N07BB99 |
| **Thyroid disorder**  (Dichotomous: 1= yes, 0 = no) | Hospital records | **Records from year prior to LMP to birth:**  E00-E07, E89.0 |
|  | Pharmaceutical  dispensing data | **Records from year prior to LMP:**  H03AA01 - H03AA02, H03BA02, H03BA03, H03BB01 |
| **Cardiovascular**  (Dichotomous: 1= yes, 0 = no) | Hospital records | **Records from year prior to LMP to birth:**  I00-I02, I05-I09, I20, I21-I24, I25, I26, I27, I28, I30-I33, I34-I39, I40-I43, I44-I49, I50, I51-I52, I60-I64, I65, I66, I67.2, I70, I73, I74, I77, G45.8, G45.9, Q20-Q25, O99.4  Supplementary: U82.1, U82.2 |
|  | Pharmaceutical  dispensing data | **Records from year prior to LMP to birth:**  C01AA05, C01BA01–C01BD01, C03, C07, C08, C09 |
| **Hypertension (pre-existing)** (Dichotomous: 1= yes, 0 = no) | Perinatal registry | **Captured at birth:**  Tick box item regarding any pre-existing  hypertension completed for all women at the time  of birth |
|  | Hospital records | **Records from year prior to LMP to birth:**  I10, I11, I12, I13, I15, O10  supplementary code: U82.3 |
|  | Pharmaceutical  dispensing data | **Records from** **year prior to LMP:**  C03AA01–C03BA11, C03DB01, C03DB99, C03EA01, C09BA02–C09BA09, C09DA02–C09DA08, C02AB01–C02AC05, C02DB02–C02DB99 (C03CA01–C03CCO1 or C09CA01–C09CX99) |
| **Diabetes (pre-existing)** (Dichotomous: 1= yes, 0 = no) | Perinatal registry | **Captured at birth:**  Tick box item regarding any pre-existing diabetes  completed for all women at the time of birth |
|  | Hospital records | **Records from year prior to LMP to birth:**  E10, E11, E13, E14, O24.0, O24.1, O24.2, O24.3 |
|  | Pharmaceutical  dispensing data | **Records from year prior to LMP:**  A10B, A10A |
| **Epilepsy**  (Dichotomous: 1= yes, 0 = no) | Hospital records | **Records from LMP to birth:**  G40, G41, F80.3, R56.1  Supplementary code: U80.3 |
|  | Pharmaceutical  dispensing data | **Records from LMP to birth:**  N03AA01 - N03AX99 |
| **Chronic renal disease**  (Dichotomous: 1= yes, 0 = no) | Hospital records | **Records from year prior to LMP to birth:**  N02-N08, N10-N12, N14-N16, N18-N19, N25-N28, Q60-Q63, N39.1, N39.2, T82.4, T86.1, Z49, Z94.0, Z99.2  supplementary code: U87.1  **Records from year prior to LMP:**  N00, N01, N17 |
|  | Pharmaceutical  dispensing data | **Records from year prior to LMP to birth:**  A11CC01 - A11CC04, B03XA01 - B03XA03, V03AE02, V03AE03, V03AE05 |
| **Mental health**  (Dichotomous: 1= yes, 0 = no) | Hospital records | **Records from year prior to LMP to birth:**  F20-F25, F28-F34, F38-F41, F43, F44, F48, F90  supplementary code: U79.2, U79.3 |
|  | Pharmaceutical  dispensing data | **Records from year prior to LMP to birth:**  N05, N06 |
|  | Medicare health services | **Records from year prior to LMP to birth:**  See [3] for full list of MBS codes. |
| **Haematological disease (Anaemia and coagulation)** | Hospital records | **Records from year prior to LMP to birth:**  D56-D57, D65-D68, D50-D53, D55, D58-D64 |
| (Dichotomous: 1= yes, 0 = no) | Pharmaceutical  dispensing data | **Records from year prior to LMP to birth:**  B01AA03 - B01AB06, B01AC04–B01AC30, B01AE07, B01AF01, B01AF02, B01AX05  PBS items: 05030R, 05035B, 05042J, 10111E, 10117L, 10129D, 10130E, 05751Q, 06456T |
| **Painful and inflammatory conditions**  (Dichotomous: 1= yes, 0 = no) | Hospital records | **Records from year prior to LMP to birth:**  D68.61, M30-M36,  Supplementary: U78.2, U80.2, U83.4, U84.1, U84.2, U86.1, U86.3 |
|  | Pharmaceutical  dispensing data | **Records from year prior to LMP to birth:**  H02AB01 - H02AB10, L04AA06, L04AA10, L04AA18, L04AD01, L04AD02, M01AB01–M01AH06 |
| **Infertility**  (Dichotomous: 1= yes, 0 = no) | Hospital records | **Records from year prior to LMP to birth:**  N97 |
| **Suspected teratogens**  (Dichotomous: 1= yes, 0 = no) | Pharmaceutical  dispensing data | **From 90 days prior to LMP to 20 weeks after LMP**  See [4] for list of ATC codes. |

ATC, Anatomical Therapeutic Chemical Classification; ICD-10-AM, International Classification of Diseases-10-Australian Modification; LMP, last menstrual period; Q, quintile.

Figure S2: Crude cumulative incidence curves for each outcome by exposure to analgesic opioids


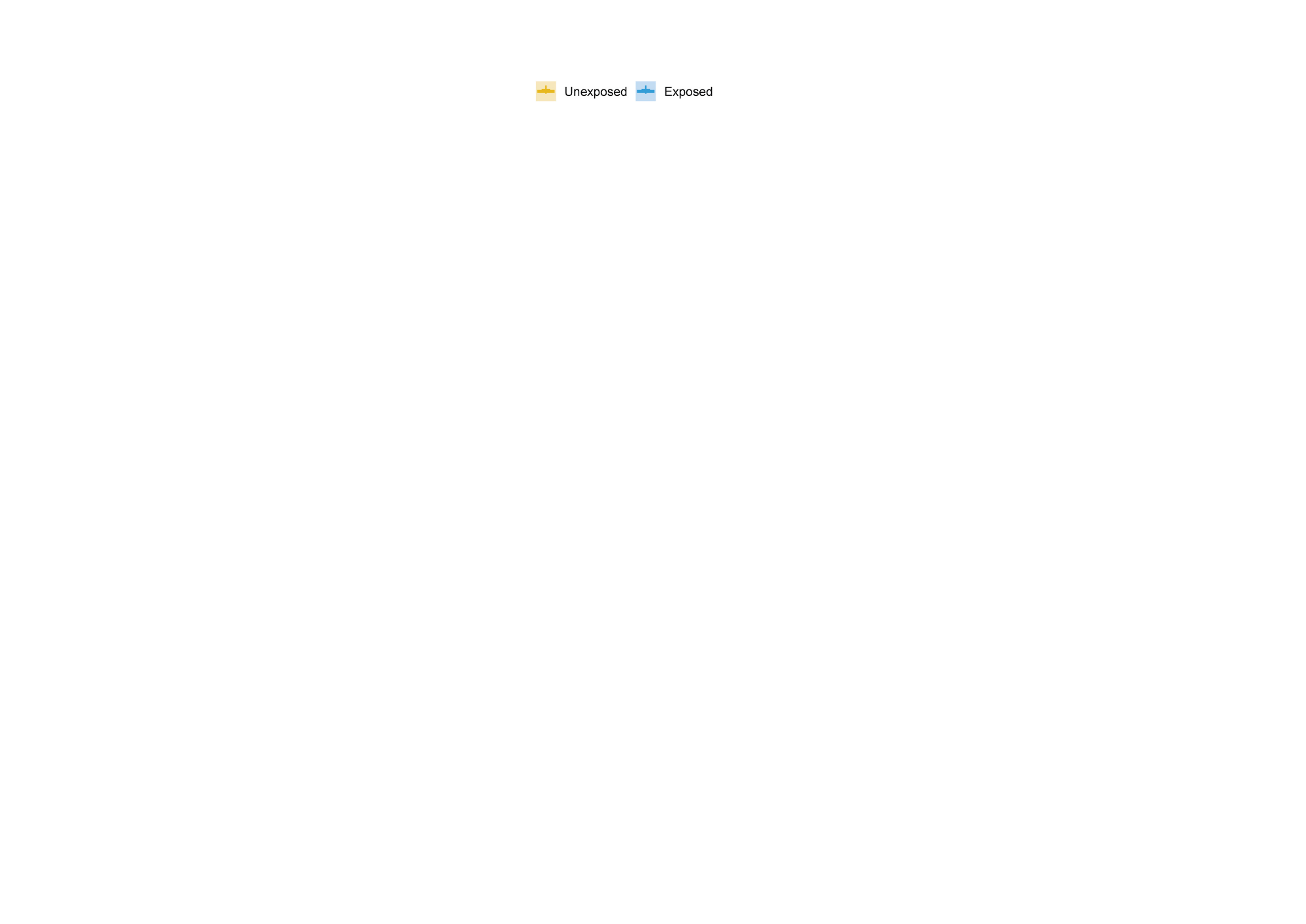

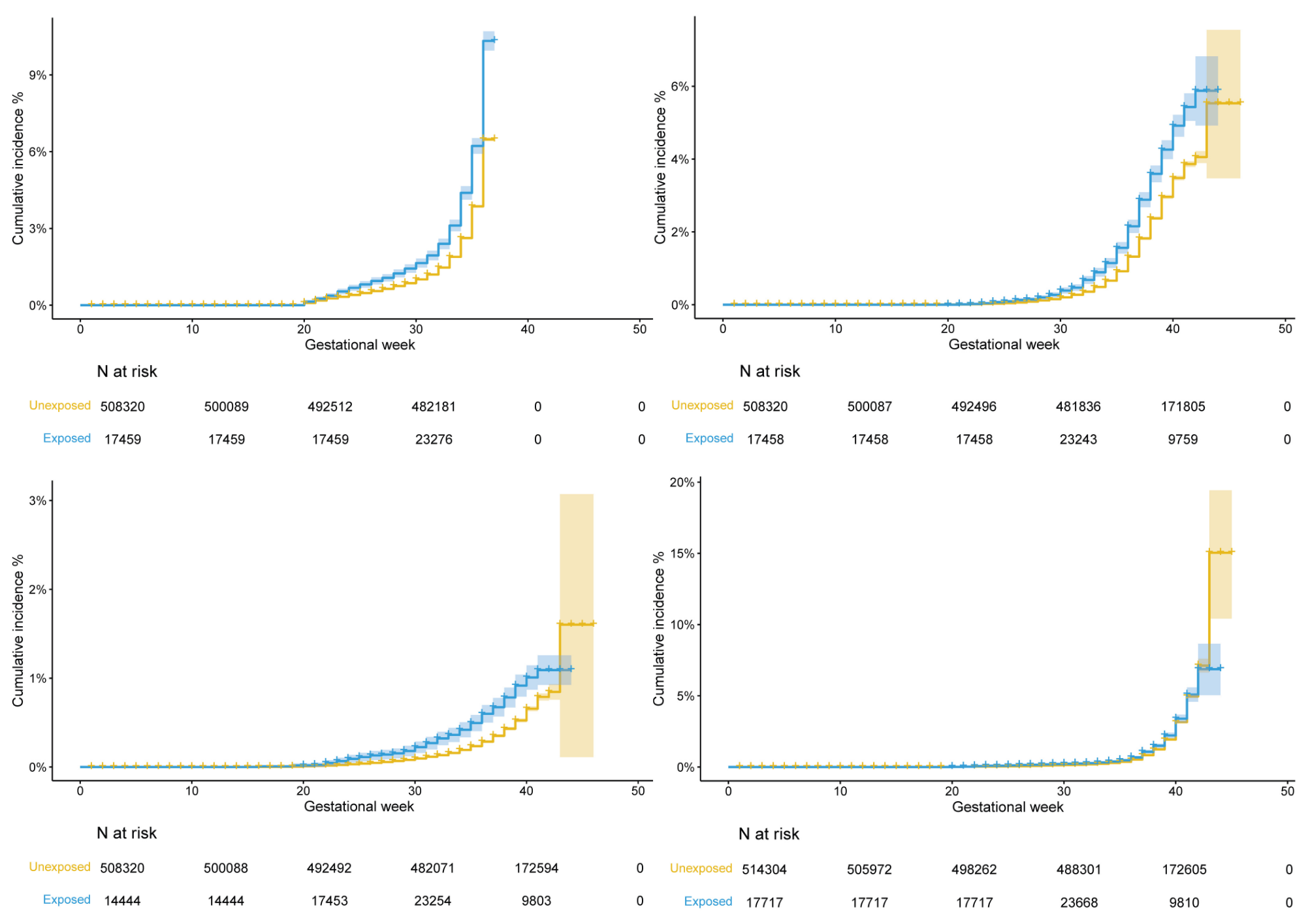


a. Preterm birth

1. Preeclampsia
2. Placental abruption

d. Fetal Growth Restriction

Table S4: Risk of preeclampsia, placental abruption, preterm birth and fetal growth restriction by opioid type of opioid and timing of exposure

|  | Events in unexposed | Events in exposed | Crude Hazard Ratio (95% CI) | Adjusted Hazard Ratio (95% CI) |
| --- | --- | --- | --- | --- |
| **Preterm** |  |  |  |  |
| Any pregnancy exposure ^a^ | 31,338 | 2733 | 1.63 (1.56-1.69) | 1.23 (1.18-1.28) |
| Codeine monotherapy | 31,338 | 2020 | 1.52 (1.45-1.59) | 1.20 (1.15-1.26) |
| Oxycodone monotherapy | 31,338 | 313 | 1.79 (1.60-2.00) | 1.27 (1.14-1.43) |
| Early pregnancy exposure ^b^ | 32,328 | 1743 | 1.54 (1.47-1.62) | 1.14 (1.09-1.20) |
| Codeine monotherapy | 32,328 | 1211 | 1.41 (1.33-1.49) | 1.11 (1.05-1.18) |
| Oxycodone monotherapy | 32,328 | 199 | 1.67 (1.45-1.92) | 1.18 (1.03-1.36) |
| Late pregnancy exposure only ^c^ | 31,338 | 990 | 1.75 (1.64-1.86) | 1.36 (1.27-1.45) |
| Codeine monotherapy | 31,338 | 809 | 1.68 (1.57-1.80) | 1.34 (1.25-1.44) |
| Oxycodone monotherapy | 31,338 | 114 | 2.05 (1.70-2.46) | 1.51 (1.25-1.81) |
| Early and late pregnancy exposure ^d^ | 31,338 | 417 | 2.24 (2.03-2.46) | 1.28 (1.16-1.41) |
| Codeine monotherapy | 31,338 | 194 | 1.80 (1.56-2.07) | 1.16 (1.01-1.34) |
| Oxycodone monotherapy | 31,338 | 28 | 2.73 (1.88-3.95) | 1.58 (1.09-2.29) |
| **Preeclampsia** |  |  |  |  |
| Any pregnancy exposure | 13,927 | 1136 | 1.45 (1.36-1.54) | 1.06 (0.99-1.13) |
| Codeine monotherapy | 13,917 | 853 | 1.36 (1.27-1.45) | 1.04 (0.97-1.11) |
| Oxycodone monotherapy | 13,868 | 131 | 1.67 (1.40-1.98) | 1.09 (0.92-1.30) |
| Early pregnancy exposure | 14,375 | 688 | 1.43 (1.32-1.54) | 1.01 (0.93-1.09) |
| Codeine monotherapy | 14,375 | 476 | 1.29 (1.18-1.41) | 0.96 (0.88-1.06) |
| Oxycodone monotherapy | 14,374 | 83 | 1.66 (1.34-2.06) | 1.07 (0.86-1.33) |
| Late pregnancy exposure only | 13,926 | 448 | 1.46 (1.33-1.60) | 1.13 (1.02-1.24) |
| Codeine monotherapy | 13,916 | 377 | 1.43 (1.29-1.58) | 1.13 (1.02-1.25) |
| Oxycodone monotherapy | 13,868 | 48 | 1.67 (1.26-2.21) | 1.13 (0.85-1.50) |
| Early and late pregnancy exposure | 13,875 | 160 | 1.90 (1.63-2.22) | 1.04 (0.89-1.22) |
| Codeine monotherapy | 13,867 | 89 | 1.76 (1.42-2.16) | 1.07 (0.87-1.33) |
| Oxycodone monotherapy | 13,862 | 13 | 3.09 (1.79-5.32) | 1.69 (0.98-2.92) |
| **Placental Abruption** |  |  |  |  |
| Any pregnancy exposure | 2602 | 227 | 1.61 (1.40-1.84) | 1.22 (1.06-1.41) |
| Codeine monotherapy | 2597 | 159 | 1.41 (1.20-1.65) | 1.13 (0.96-1.33) |
| Oxycodone monotherapy | 2590 | 31 | 2.18 (1.53-3.10) | 1.52 (1.06-2.17) |
| Early pregnancy exposure | 2673 | 156 | 1.74 (1.48-2.04) | 1.29 (1.09-1.52) |
| Codeine monotherapy | 2673 | 101 | 1.47 (1.20-1.79) | 1.17 (0.96-1.43) |
| Oxycodone monotherapy | 2670 | 22 | 2.35 (1.54-3.58) | 1.59 (1.04-2.42) |
| Late pregnancy exposure only | 2599 | 71 | 1.35 (1.07-1.71) | 1.09 (0.86-1.39) |
| Codeine monotherapy | 2594 | 58 | 1.29 (0.99-1.67) | 1.06 (0.81-1.37) |
| Oxycodone monotherapy | 2590 | 9 | 1.84 (0.96-3.54) | 1.38 (0.71-2.65) |
| Early and late pregnancy exposure | 2593 | 44 | 3.00 (2.23-4.04) | 1.76 (1.30-2.40) |
| Codeine monotherapy | 2590 | 23 | 2.62 (1.74-3.96) | 1.75 (1.16-2.66) |
| **Fetal growth restriction ^e^** |  |  |  |  |
| Any pregnancy exposure | 11,210 | 699 | 1.09 (1.01-1.18) | 0.95 (0.88-1.02) |
| Codeine monotherapy | 11,210 | 543 | 1.04 (0.95-1.13) | 0.93 (0.85-1.01) |
| Oxycodone monotherapy | 11,210 | 75 | 1.20 (0.96-1.51) | 1.08 (0.86-1.35) |
| Early pregnancy exposure | 11,467 | 442 | 1.20 (1.09-1.32) | 1.01 (0.92-1.11) |
| Codeine monotherapy | 11,467 | 327 | 1.15 (1.03-1.28) | 0.99 (0.89-1.11) |
| Oxycodone monotherapy | 11,467 | 50 | 1.32 (1.00-1.74) | 1.18 (0.89-1.56) |
| Late pregnancy exposure only | 11,210 | 257 | 0.94 (0.83-1.07) | 0.86 (0.76-0.98) |
| Codeine monotherapy | 11,210 | 216 | 0.91 (0.80-1.04) | 0.85 (0.74-0.97) |
| Oxycodone monotherapy | 11,210 | 25 | 1.02 (0.69-1.52) | 0.94 (0.63-1.39) |
| Early and late pregnancy exposure | 11,210 | 94 | 1.47 (1.20-1.80) | 1.08 (0.88-1.32) |
| Codeine monotherapy | 11,210 | 51 | 1.28 (0.97-1.68) | 1.04 (0.79-1.37) |
| Oxycodone monotherapy | 11,210 | 7 | 2.34 (1.12-4.92) | 1.97 (0.94-4.13) |
| All analyses were conducted using a time-varying exposure. | | | | |
| ^a^477,705 pregnancies had no opioid dispensings from 90 days prior to the LMP through birth. 32,266 pregnancies had at least 1 opioid dispensing within LMP to birth. Of these exposed in pregnancy 25,925 had only codeine dispensed and 3,234 had only oxycodone dispensed. | | | | |
| ^b^492,420 pregnancies had no opioid dispensings from 90 days prior to the LMP through 20 weeks after the LMP. 17,551 pregnancies had at least 1 opioid dispensing within 20 weeks after the LMP. Of these exposed only in early pregnancy 13,266 had only codeine dispensed and 1860 had only oxycodone dispensed. | | | | |
| ^c^477,705 pregnancies had no opioid dispensings from 90 days prior to the LMP through birth. 14,715 pregnancies had no opioid dispensing within 20 weeks after the LMP and at least 1 opioid dispensing between the 140th day after LMP and birth. Of these exposed only in late pregnancy 12,659 had only codeine dispensed and 1374 had only oxycodone dispensed. | | | | |
| ^d^477,705 pregnancies had no opioid dispensings from 90 days prior to the LMP through birth. 3680 pregnancies had at least 1 opioid dispensing within 20 weeks after after the LMP and at least 1 opioid dispensing between the 140th day after LMP and birth. Of these exposed in both early and late pregnancy, 2,168 had only codeine dispensed and 191 had only oxycodone dispensed. | | | | |
| ^e^Analysis based on number of infants  CI, confidence interval | | | | |

Table S5: Risk of preeclampsia, placental abruption, preterm birth and fetal growth restriction by opioid type of opioid and timing of exposure. Requiring two prescription fills in each pregnancy period.

|  | Events in unexposed | Events in exposed | Crude Hazard Ratio (95% CI) | Adjusted Hazard Ratio (95% CI) |
| --- | --- | --- | --- | --- |
| **Preterm birth** |  |  |  |  |
| Any pregnancy exposure ^a^ | 33,242 | 829 | 2.11 (1.97-2.26) | 1.31 (1.22-1.40) |
| Codeine monotherapy | 31,338 | 413 | 1.88 (1.71-2.07) | 1.26 (1.14-1.39) |
| Oxycodone monotherapy | 31,338 | 69 | 2.31 (1.82-2.93) | 1.37 (1.08-1.74) |
| Early pregnancy exposure ^b^ | 33,596 | 475 | 2.09 (1.91-2.29) | 1.23 (1.12-1.35) |
| Codeine monotherapy | 32,328 | 192 | 1.82 (1.58-2.10) | 1.20 (1.04-1.39) |
| Oxycodone monotherapy | 32,328 | 45 | 2.34 (1.75-3.14) | 1.32 (0.99-1.77) |
| Late pregnancy exposure only ^c^ | 31,338 | 167 | 2.31 (1.99-2.69) | 1.51 (1.29-1.76) |
| Codeine monotherapy | 31,338 | 101 | 2.21 (1.82-2.68) | 1.57 (1.29-1.91) |
| Oxycodone monotherapy | 31,338 | 15 | 2.36 (1.42-3.91) | 1.54 (0.93-2.56) |
| Early and late pregnancy exposure ^d^ | 31,338 | 167 | 2.76 (2.37-3.21) | 1.34 (1.15-1.57) |
| Codeine monotherapy | 31,338 | 40 | 1.96 (1.44-2.67) | 1.09 (0.80-1.48) |
| Oxycodone monotherapy | 31,338 | 15 | 3.20 (1.93-5.30) | 1.53 (0.92-2.54) |
| **Preeclampsia** |  |  |  |  |
| Any pregnancy exposure | 14,747 | 316 | 1.78 (1.59-1.99) | 1.09 (0.97-1.22) |
| Codeine monotherapy | 13,881 | 166 | 1.61 (1.38-1.88) | 1.08 (0.92-1.26) |
| Oxycodone monotherapy | 13,864 | 25 | 1.97 (1.33-2.92) | 1.01 (0.68-1.50) |
| Early pregnancy exposure | 14,876 | 187 | 2.02 (1.75-2.33) | 1.12 (0.97-1.30) |
| Codeine monotherapy | 14,374 | 78 | 1.78 (1.43-2.22) | 1.10 (0.88-1.38) |
| Oxycodone monotherapy | 14,374 | 15 | 1.93 (1.16-3.19) | 0.84 (0.51-1.40) |
| Late pregnancy exposure only | 13,881 | 58 | 1.46 (1.13-1.90) | 1.09 (0.84-1.41) |
| Codeine monotherapy | 13,876 | 38 | 1.46 (1.06-2.00) | 1.15 (0.84-1.59) |
| Early and late pregnancy exposure | 13,866 | 62 | 2.41 (1.88-3.09) | 1.08 (0.84-1.39) |
| Codeine monotherapy | 13,863 | 24 | 2.65 (1.78-3.96) | 1.21 (0.81-1.82) |
| Oxycodone monotherapy | 13,862 | 7 | 3.73 (1.78-7.82) | 1.40 (0.67-2.95) |
| **Placental abruption** |  |  |  |  |
| Any pregnancy exposure | 2749 | 80 | 2.53 (2.03-3.16) | 1.59 (1.26-2.00) |
| Codeine monotherapy | 2592 | 41 | 2.25 (1.66-3.07) | 1.54 (1.12-2.10) |
| Oxycodone monotherapy | 2587 | 6 | 2.62 (1.17-5.83) | 1.44 (0.65-3.23) |
| Early pregnancy exposure | 2782 | 47 | 2.69 (2.02-3.59) | 1.56 (1.16-2.10) |
| Codeine monotherapy | 2671 | 18 | 2.20 (1.38-3.50) | 1.46 (0.92-2.33) |
| Late pregnancy exposure only | 2590 | 13 | 1.98 (1.15-3.41) | 1.40 (0.81-2.42) |
| Codeine monotherapy | 2588 | 8 | 1.85 (0.92-3.70) | 1.35 (0.67-2.70) |
| Early and late pregnancy exposure | 2588 | 23 | 5.16 (3.42-7.78) | 2.52 (1.66-3.85) |
| Codeine monotherapy | 2587 | 7 | 4.54 (2.16-9.54) | 2.63 (1.25-5.55) |
| **Fetal growth restriction ^e^** |  |  |  |  |
| Any pregnancy exposure | 11,727 | 182 | 1.34 (1.15-1.55) | 1.03 (0.89-1.19) |
| Codeine monotherapy | 11,210 | 94 | 1.14 (0.93-1.39) | 0.94 (0.77-1.16) |
| Oxycodone monotherapy | 11,210 | 17 | 1.77 (1.10-2.86) | 1.36 (0.85-2.20) |
| Early pregnancy exposure | 11,800 | 109 | 1.63 (1.35-1.96) | 1.17 (0.97-1.42) |
| Codeine monotherapy | 11,467 | 47 | 1.44 (1.08-1.92) | 1.12 (0.84-1.49) |
| Oxycodone monotherapy | 11,467 | 11 | 1.96 (1.09-3.55) | 1.42 (0.78-2.56) |
| Late pregnancy exposure only | 11,210 | 34 | 0.99 (0.71-1.39) | 0.83 (0.59-1.16) |
| Codeine monotherapy | 11,210 | 17 | 0.73 (0.46-1.18) | 0.65 (0.41-1.05) |
| Early and late pregnancy exposure | 11,210 | 42 | 2.31 (1.71-3.13) | 1.43 (1.06-1.95) |
| Codeine monotherapy | 11,210 | 12 | 1.78 (1.01-3.14) | 1.24 (0.70-2.18) |
| All analyses were conducted using a time-varying exposure. | | | | |
| ^a^Required at least 2 opioid dispensings within LMP to birth. | | | | |
| ^b^Required at least 2 opioid dispensings within 20 weeks after the LMP. | | | | |
| ^c^Required no opioid dispensing within 20 weeks after the LMP and at least 2 opioid dispensings between the 140th day after LMP and birth. | | | | |
| ^d^Required at least 2 opioid dispensings within 20 weeks after after the LMP and at least 2 opioid dispensings between the 140th day after LMP and birth. | | | | |
| ^e^Analysis based on number of infants | | | | |

Table S6: Risk of preeclampsia, placental abruption, preterm birth and fetal growth restriction by opioid type of opioid and timing of exposure: Restricting study population to singleton births

|  | Events in unexposed | Events in exposed | Crude Hazard Ratio (95% CI) | Adjusted Hazard Ratio (95% CI) |
| --- | --- | --- | --- | --- |
| **Preterm** |  |  |  |  |
| Any pregnancy exposure | 27,335 | 2379 | 1.63 (1.56-1.70) | 1.24 (1.18-1.29) |
| Codeine monotherapy | 27,335 | 1736 | 1.50 (1.43-1.58) | 1.20 (1.14-1.26) |
| Oxycodone monotherapy | 27,335 | 283 | 1.86 (1.66-2.09) | 1.30 (1.15-1.46) |
| Early pregnancy exposure | 28,160 | 1554 | 1.58 (1.50-1.66) | 1.16 (1.10-1.22) |
| Codeine monotherapy | 28,160 | 1067 | 1.43 (1.35-1.52) | 1.13 (1.06-1.20) |
| Oxycodone monotherapy | 28,160 | 183 | 1.76 (1.53-2.04) | 1.20 (1.03-1.38) |
| Late pregnancy exposure only | 27,335 | 825 | 1.68 (1.57-1.81) | 1.35 (1.26-1.45) |
| Codeine monotherapy | 27,335 | 669 | 1.61 (1.49-1.74) | 1.30 (1.21-1.41) |
| Oxycodone monotherapy | 27,335 | 100 | 2.07 (1.70-2.52) | 1.58 (1.29-1.92) |
| Early and late pregnancy exposure | 27,335 | 379 | 2.34 (2.12-2.59) | 1.32 (1.19-1.47) |
| Codeine monotherapy | 27,335 | 175 | 1.86 (1.61-2.16) | 1.19 (1.03-1.39) |
| Oxycodone monotherapy | 27,335 | 28 | 3.14 (2.17-4.55) | 1.77 (1.22-2.57) |
| **Preeclampsia** |  |  |  |  |
| Any pregnancy exposure | 13,218 | 1075 | 1.45 (1.36-1.54) | 1.06 (1.00-1.13) |
| Codeine monotherapy | 13,208 | 811 | 1.36 (1.26-1.46) | 1.05 (0.97-1.13) |
| Oxycodone monotherapy | 13,165 | 124 | 1.66 (1.39-1.99) | 1.08 (0.91-1.29) |
| Early pregnancy exposure | 13,641 | 652 | 1.43 (1.32-1.55) | 1.02 (0.94-1.11) |
| Codeine monotherapy | 13,641 | 451 | 1.29 (1.18-1.42) | 0.99 (0.90-1.08) |
| Oxycodone monotherapy | 13,640 | 81 | 1.71 (1.38-2.13) | 1.07 (0.86-1.34) |
| Late pregnancy exposure only | 13,217 | 423 | 1.44 (1.31-1.59) | 1.12 (1.02-1.23) |
| Codeine monotherapy | 13,207 | 360 | 1.43 (1.29-1.59) | 1.12 (1.01-1.25) |
| Oxycodone monotherapy | 13,165 | 43 | 1.57 (1.16-2.11) | 1.11 (0.82-1.50) |
| Early and late pregnancy exposure | 13,171 | 150 | 1.88 (1.60-2.21) | 1.06 (0.90-1.25) |
| Codeine monotherapy | 13,164 | 83 | 1.72 (1.39-2.14) | 1.09 (0.88-1.36) |
| Oxycodone monotherapy | 13,159 | 13 | 3.28 (1.91-5.66) | 1.75 (1.01-3.02) |
| **Placental Abruption** |  |  |  |  |
| Any pregnancy exposure | 2535 | 223 | 1.62 (1.41-1.86) | 1.23 (1.07-1.42) |
| Codeine monotherapy | 2530 | 156 | 1.41 (1.20-1.66) | 1.13 (0.96-1.34) |
| Oxycodone monotherapy | 2523 | 31 | 2.24 (1.57-3.19) | 1.55 (1.09-2.22) |
| Early pregnancy exposure | 2604 | 154 | 1.77 (1.50-2.08) | 1.31 (1.11-1.55) |
| Codeine monotherapy | 2604 | 100 | 1.50 (1.23-1.83) | 1.19 (0.97-1.46) |
| Oxycodone monotherapy | 2601 | 22 | 2.42 (1.59-3.68) | 1.63 (1.07-2.48) |
| Late pregnancy exposure only | 2532 | 69 | 1.34 (1.06-1.71) | 1.09 (0.85-1.38) |
| Codeine monotherapy | 2527 | 56 | 1.27 (0.97-1.65) | 1.04 (0.80-1.36) |
| Oxycodone monotherapy | 2523 | 9 | 1.88 (0.98-3.62) | 1.41 (0.73-2.72) |
| Early and late pregnancy exposure | 2526 | 44 | 3.08 (2.28-4.15) | 1.80 (1.33-2.45) |
| Codeine monotherapy | 2523 | 23 | 2.69 (1.78-4.05) | 1.78 (1.17-2.69) |
| **Fetal growth restriction ^a^** |  |  |  |  |
| Any pregnancy exposure | 10,028 | 620 | 1.08 (1.00-1.17) | 0.97 (0.89-1.05) |
| Codeine monotherapy | 10,028 | 481 | 1.03 (0.94-1.13) | 0.94 (0.86-1.03) |
| Oxycodone monotherapy | 10,028 | 68 | 1.22 (0.96-1.55) | 1.09 (0.86-1.39) |
| Early pregnancy exposure | 10,260 | 388 | 1.19 (1.08-1.32) | 1.03 (0.93-1.14) |
| Codeine monotherapy | 10,260 | 286 | 1.13 (1.01-1.27) | 1.02 (0.90-1.14) |
| Oxycodone monotherapy | 10,260 | 45 | 1.35 (1.00-1.80) | 1.16 (0.87-1.56) |
| Late pregnancy exposure only | 10,028 | 232 | 0.94 (0.82-1.07) | 0.89 (0.78-1.01) |
| Codeine monotherapy | 10,028 | 195 | 0.90 (0.78-1.04) | 0.86 (0.74-0.99) |
| Oxycodone monotherapy | 10,028 | 23 | 1.05 (0.69-1.57) | 1.00 (0.66-1.50) |
| Early and late pregnancy exposure | 10,028 | 81 | 1.44 (1.15-1.79) | 1.07 (0.86-1.34) |
| Codeine monotherapy | 10,028 | 47 | 1.32 (0.99-1.76) | 1.08 (0.81-1.44) |
| All analyses were conducted using a time-varying exposure. | | | | |
| ^a^Analysis based on number of infants | | | | |

Table S7: Risk of preeclampsia, placental abruption, preterm birth and fetal growth restriction by opioid type of opioid and timing of exposure. Restricting to only first pregnancies

|  | Events in unexposed | Events in exposed | Crude Hazard Ratio (95% CI) | Adjusted Hazard Ratio (95% CI) |
| --- | --- | --- | --- | --- |
| **Preterm** |  |  |  |  |
| Any pregnancy exposure | 15,179 | 971 | 1.52 (1.43-1.62) | 1.19 (1.12-1.28) |
| Codeine monotherapy | 15,179 | 733 | 1.46 (1.36-1.57) | 1.18 (1.09-1.27) |
| Oxycodone monotherapy | 15,179 | 119 | 1.59 (1.33-1.91) | 1.21 (1.01-1.45) |
| Early pregnancy exposure | 15,555 | 595 | 1.36 (1.25-1.47) | 1.05 (0.97-1.14) |
| Codeine monotherapy | 15,555 | 432 | 1.29 (1.17-1.42) | 1.03 (0.93-1.13) |
| Oxycodone monotherapy | 15,555 | 71 | 1.37 (1.09-1.73) | 1.07 (0.85-1.35) |
| Late pregnancy exposure only | 15,179 | 376 | 1.84 (1.66-2.04) | 1.47 (1.32-1.63) |
| Codeine monotherapy | 15,179 | 301 | 1.77 (1.58-1.98) | 1.46 (1.30-1.63) |
| Oxycodone monotherapy | 15,179 | 48 | 2.10 (1.58-2.78) | 1.51 (1.14-2.01) |
| Early and late pregnancy exposure | 15,179 | 89 | 1.74 (1.41-2.14) | 1.06 (0.86-1.31) |
| Codeine monotherapy | 15,179 | 40 | 1.37 (1.01-1.87) | 0.95 (0.70-1.30) |
| Oxycodone monotherapy | 15,179 | 8 | 2.43 (1.22-4.86) | 1.50 (0.75-3.00) |
| **Preeclampsia** |  |  |  |  |
| Any pregnancy exposure | 8888 | 566 | 1.40 (1.29-1.53) | 0.97 (0.89-1.06) |
| Codeine monotherapy | 8883 | 431 | 1.33 (1.21-1.47) | 0.95 (0.87-1.05) |
| Oxycodone monotherapy | 8849 | 68 | 1.53 (1.21-1.94) | 0.96 (0.76-1.22) |
| Early pregnancy exposure | 9122 | 332 | 1.33 (1.20-1.49) | 0.90 (0.80-1.00) |
| Codeine monotherapy | 9122 | 236 | 1.23 (1.08-1.40) | 0.87 (0.77-1.00) |
| Oxycodone monotherapy | 9122 | 42 | 1.45 (1.07-1.97) | 0.91 (0.67-1.24) |
| Late pregnancy exposure only | 8888 | 234 | 1.49 (1.31-1.70) | 1.07 (0.94-1.22) |
| Codeine monotherapy | 8883 | 195 | 1.45 (1.26-1.67) | 1.07 (0.92-1.23) |
| Oxycodone monotherapy | 8849 | 26 | 1.66 (1.13-2.43) | 1.07 (0.73-1.57) |
| Early and late pregnancy exposure | 8850 | 53 | 1.67 (1.28-2.19) | 0.85 (0.65-1.12) |
| Codeine monotherapy | 8849 | 29 | 1.53 (1.06-2.20) | 0.89 (0.62-1.29) |
| **Placental Abruption** |  |  |  |  |
| Any pregnancy exposure | 1198 | 76 | 1.43 (1.13-1.80) | 1.17 (0.92-1.48) |
| Codeine monotherapy | 1198 | 54 | 1.26 (0.96-1.66) | 1.07 (0.81-1.41) |
| Oxycodone monotherapy | 1194 | 9 | 1.55 (0.80-2.98) | 1.16 (0.60-2.25) |
| Early pregnancy exposure | 1226 | 48 | 1.44 (1.08-1.92) | 1.15 (0.85-1.54) |
| Codeine monotherapy | 1226 | 30 | 1.17 (0.81-1.68) | 0.98 (0.68-1.41) |
| Oxycodone monotherapy | 1225 | 6 | 1.55 (0.69-3.45) | 1.13 (0.51-2.53) |
| Late pregnancy exposure only | 1197 | 28 | 1.39 (0.96-2.03) | 1.19 (0.82-1.74) |
| Codeine monotherapy | 1197 | 24 | 1.39 (0.92-2.08) | 1.21 (0.80-1.81) |
| Early and late pregnancy exposure | 1194 | 12 | 2.99 (1.69-5.28) | 1.99 (1.12-3.56) |
| **Fetal growth restriction ^a^** |  |  |  |  |
| Any pregnancy exposure | 6724 | 328 | 1.02 (0.91-1.14) | 0.92 (0.82-1.03) |
| Codeine monotherapy | 6724 | 262 | 0.99 (0.88-1.12) | 0.91 (0.80-1.03) |
| Oxycodone monotherapy | 6724 | 32 | 0.96 (0.68-1.36) | 0.91 (0.64-1.29) |
| Early pregnancy exposure | 6847 | 205 | 1.13 (0.99-1.30) | 1.00 (0.87-1.15) |
| Codeine monotherapy | 6847 | 156 | 1.11 (0.95-1.31) | 0.99 (0.84-1.16) |
| Oxycodone monotherapy | 6847 | 22 | 1.07 (0.70-1.63) | 1.07 (0.70-1.62) |
| Late pregnancy exposure only | 6724 | 123 | 0.87 (0.73-1.04) | 0.82 (0.68-0.98) |
| Codeine monotherapy | 6724 | 106 | 0.86 (0.71-1.04) | 0.82 (0.67-0.99) |
| Oxycodone monotherapy | 6724 | 10 | 0.80 (0.43-1.49) | 0.71 (0.38-1.32) |
| Early and late pregnancy exposure | 6724 | 25 | 1.05 (0.71-1.56) | 0.79 (0.53-1.18) |
| Codeine monotherapy | 6724 | 12 | 0.81 (0.46-1.43) | 0.66 (0.37-1.16) |
| All analyses were conducted using a time-varying exposure. | | | | |
| ^a^Analysis based on number of infants | | | | |

Table S8: Risk of preeclampsia, placental abruption, preterm birth and fetal growth restriction by opioid type of opioid and timing of exposure. Restricting to non-first pregnancies

|  | Events in unexposed | Events in exposed | Crude Hazard Ratio (95% CI) | adjusted Hazard Ratio (95% CI) |
| --- | --- | --- | --- | --- |
| **Preterm** |  |  |  |  |
| Any pregnancy exposure | 16,159 | 1762 | 1.74 (1.65-1.83) | 1.26 (1.19-1.32) |
| Codeine monotherapy | 16,159 | 1287 | 1.60 (1.51-1.70) | 1.22 (1.15-1.30) |
| Oxycodone monotherapy | 16,159 | 194 | 1.97 (1.71-2.27) | 1.33 (1.16-1.54) |
| Early pregnancy exposure | 16,773 | 1148 | 1.71 (1.61-1.81) | 1.21 (1.14-1.29) |
| Codeine monotherapy | 16,773 | 779 | 1.53 (1.42-1.64) | 1.18 (1.09-1.27) |
| Oxycodone monotherapy | 16,773 | 128 | 1.93 (1.62-2.29) | 1.27 (1.06-1.51) |
| Late pregnancy exposure only | 16,159 | 614 | 1.75 (1.61-1.89) | 1.30 (1.20-1.41) |
| Codeine monotherapy | 16,159 | 508 | 1.69 (1.54-1.84) | 1.28 (1.17-1.40) |
| Oxycodone monotherapy | 16,159 | 66 | 2.05 (1.61-2.61) | 1.52 (1.19-1.94) |
| Early and late pregnancy exposure | 16,159 | 328 | 2.55 (2.28-2.84) | 1.37 (1.23-1.54) |
| Codeine monotherapy | 16,159 | 154 | 2.05 (1.75-2.41) | 1.27 (1.08-1.49) |
| Oxycodone monotherapy | 16,159 | 20 | 2.98 (1.92-4.62) | 1.55 (1.00-2.40) |
| **Preeclampsia** |  |  |  |  |
| Any pregnancy exposure | 5039 | 570 | 1.74 (1.60-1.90) | 1.18 (1.07-1.29) |
| Codeine monotherapy | 5034 | 422 | 1.61 (1.46-1.78) | 1.14 (1.03-1.26) |
| Oxycodone monotherapy | 5019 | 63 | 2.03 (1.58-2.60) | 1.29 (1.01-1.66) |
| Early pregnancy exposure | 5253 | 356 | 1.76 (1.58-1.96) | 1.15 (1.02-1.28) |
| Codeine monotherapy | 5253 | 240 | 1.55 (1.36-1.77) | 1.08 (0.95-1.24) |
| Oxycodone monotherapy | 5252 | 41 | 2.08 (1.53-2.83) | 1.34 (0.99-1.83) |
| Late pregnancy exposure only | 5038 | 214 | 1.67 (1.46-1.91) | 1.19 (1.04-1.37) |
| Codeine monotherapy | 5033 | 182 | 1.65 (1.42-1.91) | 1.20 (1.03-1.39) |
| Oxycodone monotherapy | 5019 | 22 | 1.90 (1.25-2.89) | 1.20 (0.79-1.83) |
| Early and late pregnancy exposure | 5025 | 107 | 2.64 (2.18-3.19) | 1.20 (0.98-1.46) |
| Codeine monotherapy | 5018 | 60 | 2.46 (1.91-3.17) | 1.20 (0.93-1.56) |
| Oxycodone monotherapy | 5015 | 8 | 4.00 (2.00-8.00) | 2.22 (1.11-4.45) |
| **Placental Abruption** |  |  |  |  |
| Any pregnancy exposure | 1404 | 151 | 1.72 (1.46-2.04) | 1.26 (1.06-1.50) |
| Codeine monotherapy | 1399 | 105 | 1.50 (1.23-1.83) | 1.16 (0.95-1.42) |
| Oxycodone monotherapy | 1396 | 22 | 2.63 (1.73-4.01) | 1.78 (1.16-2.72) |
| Early pregnancy exposure | 1447 | 108 | 1.92 (1.58-2.34) | 1.38 (1.13-1.69) |
| Codeine monotherapy | 1447 | 71 | 1.66 (1.31-2.10) | 1.28 (1.01-1.63) |
| Oxycodone monotherapy | 1445 | 16 | 2.92 (1.78-4.78) | 1.92 (1.17-3.15) |
| Late pregnancy exposure only | 1402 | 43 | 1.33 (0.98-1.81) | 1.04 (0.76-1.41) |
| Codeine monotherapy | 1397 | 34 | 1.23 (0.87-1.73) | 0.97 (0.69-1.36) |
| Oxycodone monotherapy | 1396 | 6 | 2.06 (0.92-4.60) | 1.50 (0.67-3.36) |
| Early and late pregnancy exposure | 1399 | 32 | 3.03 (2.14-4.31) | 1.74 (1.21-2.50) |
| Codeine monotherapy | 1396 | 18 | 2.86 (1.80-4.55) | 1.84 (1.15-2.95) |
| **Fetal growth restriction ^a^** |  |  |  |  |
| Any pregnancy exposure | 4486 | 371 | 1.27 (1.14-1.41) | 0.95 (0.85-1.06) |
| Codeine monotherapy | 4486 | 281 | 1.18 (1.05-1.34) | 0.93 (0.82-1.05) |
| Oxycodone monotherapy | 4486 | 43 | 1.56 (1.16-2.11) | 1.24 (0.92-1.68) |
| Early pregnancy exposure | 4620 | 237 | 1.38 (1.22-1.58) | 1.00 (0.88-1.15) |
| Codeine monotherapy | 4620 | 171 | 1.29 (1.11-1.50) | 0.99 (0.85-1.15) |
| Oxycodone monotherapy | 4620 | 28 | 1.69 (1.17-2.45) | 1.28 (0.88-1.86) |
| Late pregnancy exposure only | 4486 | 134 | 1.10 (0.93-1.31) | 0.89 (0.75-1.06) |
| Codeine monotherapy | 4486 | 110 | 1.05 (0.87-1.26) | 0.86 (0.71-1.04) |
| Oxycodone monotherapy | 4486 | 15 | 1.37 (0.82-2.27) | 1.20 (0.72-2.00) |
| Early and late pregnancy exposure | 4486 | 69 | 2.01 (1.59-2.55) | 1.18 (0.93-1.51) |
| Codeine monotherapy | 4486 | 39 | 1.83 (1.33-2.51) | 1.21 (0.88-1.66) |
| All analyses were conducted using a time-varying exposure. | | | | |
| ^a^Analysis based on number of infants | | | | |

Table S9: Risk of preeclampsia, placental abruption, preterm birth and fetal growth restriction by opioid type of opioid and timing of exposure. Restricting covariates included in the regression model to those only measured before pregnancy

|  | Events in unexposed | Events in exposed | Crude Hazard Ratio (95% CI) | adjusted Hazard Ratio (95% CI) |
| --- | --- | --- | --- | --- |
| **Preterm** |  |  |  |  |
| Any pregnancy exposure | 31,338 | 2733 | 1.63 (1.56-1.69) | 1.28 (1.23-1.33) |
| Codeine monotherapy | 31,338 | 2020 | 1.52 (1.45-1.59) | 1.24 (1.18-1.30) |
| Oxycodone monotherapy | 31,338 | 313 | 1.79 (1.60-2.00) | 1.36 (1.21-1.52) |
| Early pregnancy exposure | 32,328 | 1743 | 1.54 (1.47-1.62) | 1.19 (1.14-1.25) |
| Codeine monotherapy | 32,328 | 1211 | 1.41 (1.33-1.49) | 1.14 (1.08-1.21) |
| Oxycodone monotherapy | 32,328 | 199 | 1.67 (1.45-1.92) | 1.25 (1.08-1.44) |
| Late pregnancy exposure only | 31,338 | 990 | 1.75 (1.64-1.86) | 1.41 (1.33-1.51) |
| Codeine monotherapy | 31,338 | 809 | 1.68 (1.57-1.80) | 1.39 (1.29-1.49) |
| Oxycodone monotherapy | 31,338 | 114 | 2.05 (1.70-2.46) | 1.62 (1.35-1.95) |
| Early and late pregnancy exposure | 31,338 | 417 | 2.24 (2.03-2.46) | 1.39 (1.26-1.54) |
| Codeine monotherapy | 31,338 | 194 | 1.80 (1.56-2.07) | 1.23 (1.07-1.42) |
| Oxycodone monotherapy | 31,338 | 28 | 2.73 (1.88-3.95) | 1.66 (1.15-2.41) |
| **Preeclampsia** |  |  |  |  |
| Any pregnancy exposure | 13,927 | 1136 | 1.45 (1.36-1.54) | 1.20 (1.12-1.27) |
| Codeine monotherapy | 13,917 | 853 | 1.36 (1.27-1.45) | 1.15 (1.08-1.24) |
| Oxycodone monotherapy | 13,868 | 131 | 1.67 (1.40-1.98) | 1.28 (1.08-1.52) |
| Early pregnancy exposure | 14,375 | 688 | 1.43 (1.32-1.54) | 1.14 (1.05-1.23) |
| Codeine monotherapy | 14,375 | 476 | 1.29 (1.18-1.41) | 1.07 (0.97-1.17) |
| Oxycodone monotherapy | 14,374 | 83 | 1.66 (1.34-2.06) | 1.23 (0.99-1.53) |
| Late pregnancy exposure only | 13,926 | 448 | 1.46 (1.33-1.60) | 1.27 (1.16-1.40) |
| Codeine monotherapy | 13,916 | 377 | 1.43 (1.29-1.58) | 1.27 (1.14-1.40) |
| Oxycodone monotherapy | 13,868 | 48 | 1.67 (1.26-2.21) | 1.36 (1.02-1.80) |
| Early and late pregnancy exposure | 13,875 | 160 | 1.90 (1.63-2.22) | 1.31 (1.11-1.53) |
| Codeine monotherapy | 13,867 | 89 | 1.76 (1.42-2.16) | 1.30 (1.05-1.60) |
| Oxycodone monotherapy | 13,862 | 13 | 3.09 (1.79-5.32) | 1.89 (1.09-3.25) |
| **Placental Abruption** |  |  |  |  |
| Any pregnancy exposure | 2602 | 227 | 1.61 (1.40-1.84) | 1.28 (1.11-1.47) |
| Codeine monotherapy | 2597 | 159 | 1.41 (1.20-1.65) | 1.16 (0.99-1.37) |
| Oxycodone monotherapy | 2590 | 31 | 2.18 (1.53-3.10) | 1.66 (1.16-2.37) |
| Early pregnancy exposure | 2673 | 156 | 1.74 (1.48-2.04) | 1.34 (1.14-1.58) |
| Codeine monotherapy | 2673 | 101 | 1.47 (1.20-1.79) | 1.20 (0.98-1.46) |
| Oxycodone monotherapy | 2670 | 22 | 2.35 (1.54-3.58) | 1.73 (1.14-2.64) |
| Late pregnancy exposure only | 2599 | 71 | 1.35 (1.07-1.71) | 1.15 (0.90-1.45) |
| Codeine monotherapy | 2594 | 58 | 1.29 (0.99-1.67) | 1.10 (0.85-1.43) |
| Oxycodone monotherapy | 2590 | 9 | 1.84 (0.96-3.54) | 1.50 (0.78-2.90) |
| Early and late pregnancy exposure | 2593 | 44 | 3.00 (2.23-4.04) | 1.89 (1.39-2.57) |
| Codeine monotherapy | 2590 | 23 | 2.62 (1.74-3.96) | 1.83 (1.21-2.76) |
| **Fetal growth restriction ^a^** |  |  |  |  |
| Any pregnancy exposure | 11,210 | 699 | 1.09 (1.01-1.18) | 0.96 (0.89-1.04) |
| Codeine monotherapy | 11,210 | 543 | 1.04 (0.95-1.13) | 0.94 (0.86-1.02) |
| Oxycodone monotherapy | 11,210 | 75 | 1.20 (0.96-1.51) | 1.09 (0.87-1.37) |
| Early pregnancy exposure | 11,467 | 442 | 1.20 (1.09-1.32) | 1.02 (0.93-1.13) |
| Codeine monotherapy | 11,467 | 327 | 1.15 (1.03-1.28) | 1.00 (0.90-1.12) |
| Oxycodone monotherapy | 11,467 | 50 | 1.32 (1.00-1.74) | 1.19 (0.90-1.58) |
| Late pregnancy exposure only | 11,210 | 257 | 0.94 (0.83-1.07) | 0.88 (0.77-0.99) |
| Codeine monotherapy | 11,210 | 216 | 0.91 (0.80-1.04) | 0.86 (0.75-0.98) |
| Oxycodone monotherapy | 11,210 | 25 | 1.02 (0.69-1.52) | 0.95 (0.64-1.40) |
| Early and late pregnancy exposure | 11,210 | 94 | 1.47 (1.20-1.80) | 1.12 (0.91-1.37) |
| Codeine monotherapy | 11,210 | 51 | 1.28 (0.97-1.68) | 1.05 (0.80-1.39) |
| Oxycodone monotherapy | 11,210 | 7 | 2.34 (1.12-4.92) | 2.06 (0.98-4.34) |
| All analyses were conducted using a time-varying exposure. | | | | |
| ^a^Analysis based on number of infants | | | | |

Table S10: Risk of preeclampsia, placental abruption, preterm birth and fetal growth restriction by opioid type of opioid and timing of exposure. Including Body Mass Index (BMI) by restricting analyses to births from 2016 onwards.

| Outcome & exposure window | Events in unexposed | Events in exposed | Crude Hazard Ratio (95% CI) | adjusted Hazard Ratio (95% CI) |
| --- | --- | --- | --- | --- |
| **Preterm** |  |  |  |  |
| Any pregnancy exposure | 19,876 | 1715 | 1.60 (1.52-1.68) | 1.23 (1.16-1.29) |
| Codeine monotherapy | 19,876 | 1249 | 1.49 (1.41-1.58) | 1.20 (1.13-1.27) |
| Oxycodone monotherapy | 19,876 | 219 | 1.81 (1.59-2.07) | 1.35 (1.18-1.54) |
| Early pregnancy exposure | 20,471 | 1120 | 1.54 (1.45-1.63) | 1.16 (1.09-1.24) |
| Codeine monotherapy | 20,471 | 771 | 1.41 (1.31-1.52) | 1.13 (1.05-1.21) |
| Oxycodone monotherapy | 20,471 | 143 | 1.76 (1.50-2.08) | 1.34 (1.13-1.58) |
| Late pregnancy exposure only | 19,876 | 595 | 1.67 (1.54-1.82) | 1.32 (1.22-1.44) |
| Codeine monotherapy | 19,876 | 478 | 1.61 (1.47-1.76) | 1.31 (1.20-1.44) |
| Oxycodone monotherapy | 19,876 | 76 | 1.90 (1.52-2.39) | 1.39 (1.11-1.74) |
| Early and late pregnancy exposure | 19,876 | 269 | 2.19 (1.94-2.47) | 1.26 (1.11-1.42) |
| Codeine monotherapy | 19,876 | 122 | 1.76 (1.47-2.10) | 1.10 (0.91-1.31) |
| Oxycodone monotherapy | 19,876 | 25 | 3.65 (2.46-5.40) | 2.44 (1.65-3.61) |
| **Preeclampsia** |  |  |  |  |
| Any pregnancy exposure | 9344 | 743 | 1.41 (1.31-1.52) | 1.02 (0.94-1.10) |
| Codeine monotherapy | 9337 | 544 | 1.30 (1.19-1.42) | 0.98 (0.90-1.07) |
| Oxycodone monotherapy | 9307 | 96 | 1.68 (1.37-2.05) | 1.09 (0.89-1.33) |
| Early pregnancy exposure | 9631 | 456 | 1.40 (1.27-1.53) | 0.99 (0.90-1.09) |
| Codeine monotherapy | 9631 | 311 | 1.25 (1.12-1.40) | 0.93 (0.83-1.04) |
| Oxycodone monotherapy | 9631 | 59 | 1.65 (1.28-2.13) | 1.08 (0.84-1.40) |
| Late pregnancy exposure only | 9344 | 287 | 1.41 (1.26-1.59) | 1.06 (0.94-1.19) |
| Codeine monotherapy | 9337 | 233 | 1.36 (1.19-1.54) | 1.04 (0.91-1.18) |
| Oxycodone monotherapy | 9307 | 37 | 1.72 (1.24-2.37) | 1.10 (0.79-1.52) |
| Early and late pregnancy exposure | 9311 | 105 | 1.81 (1.49-2.20) | 0.99 (0.81-1.20) |
| Codeine monotherapy | 9305 | 58 | 1.69 (1.31-2.19) | 0.97 (0.74-1.26) |
| Oxycodone monotherapy | 9302 | 9 | 3.05 (1.59-5.86) | 1.82 (0.94-3.50) |
| **Placental Abruption** |  |  |  |  |
| Any pregnancy exposure | 1752 | 158 | 1.66 (1.41-1.95) | 1.26 (1.06-1.49) |
| Codeine monotherapy | 1747 | 112 | 1.49 (1.23-1.80) | 1.19 (0.98-1.45) |
| Oxycodone monotherapy | 1742 | 20 | 1.92 (1.24-2.99) | 1.34 (0.86-2.10) |
| Early pregnancy exposure | 1803 | 107 | 1.74 (1.43-2.12) | 1.30 (1.06-1.58) |
| Codeine monotherapy | 1803 | 71 | 1.52 (1.20-1.93) | 1.22 (0.96-1.55) |
| Oxycodone monotherapy | 1801 | 14 | 2.07 (1.22-3.50) | 1.43 (0.84-2.43) |
| Late pregnancy exposure only | 1750 | 51 | 1.47 (1.11-1.94) | 1.17 (0.88-1.55) |
| Codeine monotherapy | 1745 | 41 | 1.40 (1.02-1.91) | 1.14 (0.83-1.55) |
| Early and late pregnancy exposure | 1744 | 32 | 3.15 (2.22-4.47) | 1.82 (1.27-2.62) |
| Codeine monotherapy | 1742 | 16 | 2.70 (1.65-4.41) | 1.75 (1.06-2.87) |
| **Fetal growth restriction ^a^** |  |  |  |  |
| Any pregnancy exposure | 7710 | 457 | 1.04 (0.95-1.14) | 0.94 (0.86-1.04) |
| Codeine monotherapy | 7710 | 355 | 1.00 (0.90-1.12) | 0.94 (0.84-1.04) |
| Oxycodone monotherapy | 7710 | 56 | 1.20 (0.92-1.56) | 1.12 (0.86-1.45) |
| Early pregnancy exposure | 7874 | 293 | 1.15 (1.02-1.29) | 1.02 (0.91-1.15) |
| Codeine monotherapy | 7874 | 222 | 1.14 (0.99-1.30) | 1.04 (0.91-1.19) |
| Oxycodone monotherapy | 7874 | 37 | 1.34 (0.97-1.84) | 1.24 (0.90-1.71) |
| Late pregnancy exposure only | 7710 | 164 | 0.89 (0.76-1.04) | 0.84 (0.72-0.99) |
| Codeine monotherapy | 7710 | 133 | 0.84 (0.71-1.00) | 0.81 (0.69-0.97) |
| Oxycodone monotherapy | 7710 | 19 | 1.02 (0.65-1.60) | 0.96 (0.61-1.50) |
| Early and late pregnancy exposure | 7710 | 55 | 1.23 (0.94-1.60) | 0.96 (0.74-1.26) |
| Codeine monotherapy | 7710 | 34 | 1.24 (0.89-1.74) | 1.06 (0.75-1.49) |
| All analyses were conducted using a time-varying exposure. | | | | |
| ^a^Analysis based on number of infants | | | | |

Table S11: Risk of preeclampsia, placental abruption, preterm birth and fetal growth restriction by opioid type of opioid and timing of exposure. Using women who had opioid exposure prior to but not during pregnancy (opioid discontinuers) as comparator.

|  | Events in unexposed | Events in exposed | Crude Hazard Ratio (95% CI) | adjusted Hazard Ratio (95% CI) |
| --- | --- | --- | --- | --- |
| **Preterm** |  |  |  |  |
| Any pregnancy exposure ^a^ | 3002 | 2733 | 1.46 (1.39-1.54) | 1.32 (1.25-1.39) |
| Codeine monotherapy | 3002 | 2020 | 1.35 (1.27-1.43) | 1.26 (1.19-1.34) |
| Oxycodone monotherapy | 3002 | 313 | 1.42 (1.26-1.60) | 1.23 (1.09-1.39) |
| Early pregnancy exposure ^b^ | 3002 | 1743 | 1.21 (1.14-1.28) | 1.08 (1.02-1.15) |
| Codeine monotherapy | 3002 | 1211 | 1.11 (1.04-1.18) | 1.04 (0.97-1.11) |
| Oxycodone monotherapy | 3002 | 199 | 1.31 (1.13-1.51) | 1.11 (0.96-1.29) |
| Late pregnancy exposure only ^c^ | 3002 | 990 | 1.55 (1.44-1.67) | 1.43 (1.33-1.54) |
| Codeine monotherapy | 3002 | 809 | 1.47 (1.36-1.59) | 1.39 (1.28-1.51) |
| Oxycodone monotherapy | 3002 | 114 | 1.62 (1.34-1.95) | 1.45 (1.20-1.75) |
| Early and late pregnancy exposure ^d^ | 3002 | 417 | 1.77 (1.60-1.97) | 1.31 (1.17-1.46) |
| Codeine monotherapy | 3002 | 194 | 1.42 (1.23-1.64) | 1.14 (0.98-1.33) |
| Oxycodone monotherapy | 3002 | 28 | 2.13 (1.47-3.09) | 1.51 (1.03-2.19) |
| **Preeclampsia** |  |  |  |  |
| Any pregnancy exposure | 1333 | 1136 | 1.24 (1.14-1.34) | 1.14 (1.05-1.24) |
| Codeine monotherapy | 1323 | 853 | 1.15 (1.06-1.26) | 1.10 (1.01-1.21) |
| Oxycodone monotherapy | 1274 | 131 | 1.34 (1.12-1.61) | 1.15 (0.96-1.38) |
| Early pregnancy exposure | 1269 | 688 | 1.14 (1.04-1.25) | 1.04 (0.94-1.14) |
| Codeine monotherapy | 1269 | 476 | 1.03 (0.93-1.15) | 0.98 (0.88-1.09) |
| Oxycodone monotherapy | 1268 | 83 | 1.32 (1.06-1.65) | 1.10 (0.88-1.38) |
| Late pregnancy exposure only | 1332 | 448 | 1.26 (1.13-1.40) | 1.22 (1.09-1.36) |
| Codeine monotherapy | 1322 | 377 | 1.23 (1.09-1.38) | 1.21 (1.08-1.36) |
| Oxycodone monotherapy | 1274 | 48 | 1.37 (1.03-1.83) | 1.21 (0.90-1.61) |
| Early and late pregnancy exposure | 1281 | 160 | 1.52 (1.29-1.80) | 1.17 (0.98-1.39) |
| Codeine monotherapy | 1273 | 89 | 1.42 (1.15-1.76) | 1.18 (0.95-1.48) |
| Oxycodone monotherapy | 1268 | 13 | 2.45 (1.42-4.23) | 1.90 (1.09-3.31) |
| **Placental Abruption** |  |  |  |  |
| Any pregnancy exposure | 238 | 227 | 1.52 (1.26-1.82) | 1.35 (1.12-1.62) |
| Codeine monotherapy | 233 | 159 | 1.32 (1.08-1.62) | 1.24 (1.01-1.52) |
| Oxycodone monotherapy | 226 | 31 | 1.89 (1.30-2.75) | 1.52 (1.03-2.22) |
| Early pregnancy exposure | 226 | 156 | 1.46 (1.19-1.79) | 1.25 (1.01-1.54) |
| Codeine monotherapy | 226 | 101 | 1.24 (0.98-1.56) | 1.14 (0.90-1.44) |
| Oxycodone monotherapy | 223 | 22 | 1.98 (1.28-3.07) | 1.53 (0.98-2.39) |
| Late pregnancy exposure only | 235 | 71 | 1.32 (1.00-1.73) | 1.22 (0.93-1.61) |
| Codeine monotherapy | 230 | 58 | 1.25 (0.93-1.68) | 1.18 (0.88-1.59) |
| Oxycodone monotherapy | 226 | 9 | 1.67 (0.86-3.26) | 1.41 (0.72-2.76) |
| Early and late pregnancy exposure | 229 | 44 | 2.65 (1.92-3.67) | 1.88 (1.33-2.66) |
| Codeine monotherapy | 226 | 23 | 2.35 (1.53-3.62) | 1.88 (1.21-2.93) |
| **Fetal growth restriction ^e^** |  |  |  |  |
| Any pregnancy exposure | 823 | 699 | 1.14 (1.03-1.26) | 1.02 (0.92-1.13) |
| Codeine monotherapy | 823 | 543 | 1.08 (0.97-1.20) | 1.00 (0.90-1.12) |
| Oxycodone monotherapy | 823 | 75 | 1.16 (0.92-1.47) | 1.04 (0.82-1.33) |
| Early pregnancy exposure | 823 | 442 | 1.14 (1.02-1.28) | 1.00 (0.89-1.13) |
| Codeine monotherapy | 823 | 327 | 1.09 (0.96-1.24) | 1.00 (0.88-1.14) |
| Oxycodone monotherapy | 823 | 50 | 1.25 (0.94-1.67) | 1.11 (0.83-1.49) |
| Late pregnancy exposure only | 823 | 257 | 0.98 (0.85-1.13) | 0.94 (0.81-1.08) |
| Codeine monotherapy | 823 | 216 | 0.95 (0.81-1.10) | 0.92 (0.79-1.07) |
| Oxycodone monotherapy | 823 | 25 | 1.01 (0.68-1.50) | 0.92 (0.62-1.38) |
| Early and late pregnancy exposure | 823 | 94 | 1.43 (1.16-1.77) | 1.08 (0.86-1.36) |
| Codeine monotherapy | 823 | 51 | 1.25 (0.94-1.65) | 1.07 (0.80-1.42) |
| Oxycodone monotherapy | 823 | 7 | 2.24 (1.06-4.72) | 1.85 (0.87-3.93) |
| All analyses were conducted using a time-varying exposure. | | | | |
| ^a^36,191 pregnancies had at least 1 opioid dispensing from 12 months to 90 days before LMP but no opioid dispensings 90 days before LMP through to birth. 32,266 pregnancies had at least 1 opioid dispensing within LMP to birth. Of these exposed in pregnancy 25,925 had only codeine dispensed and 3,234 had only oxycodone dispensed. | | | | |
| ^b^36,191 pregnancies had at least 1 opioid dispensing from 12 months to 90 days before LMP but no opioid dispensings 90 days before LMP through to birth. 17,551 pregnancies had at least 1 opioid dispensing within 140 days after the LMP. Of these exposed only in early pregnancy 13,266 had only codeine dispensed and 1860 had only oxycodone dispensed. | | | | |
| ^c^36,191 pregnancies had at least 1 opioid dispensing from 12 months to 90 days before LMP but no opioid dispensings 90 days before LMP through to birth. 14,715 pregnancies had no opioid dispensing within 140 days after the LMP and at least 1 opioid dispensing between the 140th day after LMP and birth.Of these exposed only in late pregnancy 12,659 had only codeine dispensed and 1374 had only oxycodone dispensed. | | | | |
| ^d^36,191 pregnancies had at least 1 opioid dispensing from 12 months to 90 days before LMP but no opioid dispensings 90 days before LMP through to birth. 3680 pregnancies had at least 1 opioid dispensing within 140 days after the LMP and at least 1 opioid dispensing between the 140th day after LMP and birth. Of these exposed in both early and late pregnancy, 2,168 had only codeine dispensed and 191 had only oxycodone dispensed. | | | | |
| ^e^Analysis based on number of infants | | | | |

Table S12: Risk of preeclampsia, placental abruption, preterm birth and fetal growth restriction by opioid type of opioid and timing of exposure. Including pregnant women with opioid exposure in the 90 days prior to pregnancy but not the first 20 weeks of pregnancy in the exposed group

|  | Events in unexposed | Events in exposed | Crude Hazard Ratio (95% CI) | Adjusted Hazard Ratio (95% CI) |
| --- | --- | --- | --- | --- |
| **Preterm** |  |  |  |  |
| Any pregnancy exposure | 31,338 | 4094 | 1.51 (1.46-1.56) | 1.11 (1.07-1.14) |
| Early pregnancy exposure | 32,328 | 3104 | 1.43 (1.38-1.48) | 1.03 (0.99-1.07) |
| Early and late pregnancy exposure | 31,338 | 554 | 2.24 (2.06-2.44) | 1.31 (1.20-1.43) |
| **Preeclampsia** |  |  |  |  |
| Any pregnancy exposure | 13,926 | 1720 | 1.40 (1.33-1.47) | 1.02 (0.96-1.07) |
| Early pregnancy exposure | 14,374 | 1272 | 1.36 (1.29-1.45) | 0.97 (0.92-1.03) |
| Early and late pregnancy exposure | 13,884 | 213 | 1.86 (1.63-2.14) | 1.06 (0.93-1.22) |
| **Placental Abruption** |  |  |  |  |
| Any pregnancy exposure | 2599 | 338 | 1.50 (1.34-1.68) | 1.15 (1.02-1.29) |
| Early pregnancy exposure | 2670 | 267 | 1.54 (1.35-1.74) | 1.16 (1.02-1.33) |
| Early and late pregnancy exposure | 2594 | 52 | 2.63 (2.00-3.46) | 1.58 (1.19-2.10) |
| **Fetal growth restriction ^a^** |  |  |  |  |
| Any pregnancy exposure | 11,178 | 1074 | 1.09 (1.02-1.16) | 0.95 (0.89-1.01) |
| Early pregnancy exposure | 11,435 | 817 | 1.14 (1.06-1.23) | 0.98 (0.91-1.06) |
| Early and late pregnancy exposure | 11,210 | 125 | 1.41 (1.18-1.68) | 1.06 (0.89-1.27) |
| All analyses were conducted using a time-varying exposure. | | | | |
| ^a^Analysis based on number of infants | | | | |

Table S13: Poisson regression analysis for risk of fetal growth restriction

|  | Events in unexposed | Events in exposed | Crude Risk Ratio (95% CI) | Adjusted Risk Ratio (95% CI) |
| --- | --- | --- | --- | --- |
| **Fetal growth restriction ^a^** |  |  |  |  |
| Any pregnancy exposure | 11,210 | 699 | 0.92 (0.85-0.99) | 0.85 (0.79-0.92) |
| Codeine monotherapy | 11,210 | 543 | 0.89 (0.82-0.97) | 0.84 (0.77-0.91) |
| Oxycodone monotherapy | 11,210 | 75 | 0.99 (0.79-1.24) | 0.94 (0.75-1.17) |
| Early pregnancy exposure | 11,467 | 442 | 1.08 (0.98-1.19) | 0.96 (0.87-1.06) |
| Codeine monotherapy | 11,467 | 327 | 1.06 (0.95-1.18) | 0.96 (0.86-1.07) |
| Oxycodone monotherapy | 11,467 | 50 | 1.15 (0.87-1.53) | 1.07 (0.82-1.41) |
| Late pregnancy exposure only | 11,210 | 257 | 0.74 (0.65-0.84) | 0.72 (0.64-0.82) |
| Codeine monotherapy | 11,210 | 216 | 0.72 (0.63-0.83) | 0.71 (0.62-0.81) |
| Oxycodone monotherapy | 11,210 | 25 | 0.77 (0.52-1.14) | 0.77 (0.52-1.13) |
| Early and late pregnancy exposure | 11,210 | 94 | 1.08 (0.89-1.33) | 0.94 (0.77-1.15) |
| Codeine monotherapy | 11,210 | 51 | 1.00 (0.76-1.31) | 0.93 (0.71-1.22) |
| Oxycodone monotherapy | 11,210 | 7 | 1.57 (0.69-3.58) | 1.52 (0.68-3.38) |
| All analyses were conducted using a time-varying exposure. | | | | |
| ^a^Analysis based on number of infants | | | | |

Supplementary references:

1. Jammal, W. and G. Gown, *Opioid prescribing pitfalls: medicolegal and regulatory issues.* Australian Prescriber, 2015. **38**(6): p. 198.

2. Australia Bureau of Statistics. *Standard Australian classification of countries (SACC), 2016*. 2016 [cited 2024; Available from: <https://www.abs.gov.au/statistics/classifications/standard-australian-classification-countries-sacc/latest-release>.

3. Australian Institute of Health and Welfare, *Medicare-subsidised mental health-specific services*, in *Mental health services in Australia*. 2023.

4. Raichand, S., et al., *Utilisation of teratogenic medicines before and during pregnancy in Australian women.* Aust N Z J Obstet Gynaecol, 2020. **60**(2): p. 218-224.
